# Controlling the thermally activated delayed fluorescence of axially chiral organic emitters and their racemate for information encryption†

**DOI:** 10.1039/d1sc04738h

**Published:** 2021-11-18

**Authors:** Bingjia Xu, Zicun Song, Minmin Zhang, Qingqing Zhang, Long Jiang, Cao Xu, Lijun Zhong, Changlin Su, Qiqi Ban, Cong Liu, Fengqiang Sun, Yi Zhang, Zhenguo Chi, Zujin Zhao, Guang Shi

**Affiliations:** Key Laboratory of Theoretical Chemistry of Environment, Ministry of Education, School of Chemistry, South China Normal University Guangzhou 510006 China bingjiaxu@m.scnu.edu.cn shiguang@scnu.edu.cn; State Key Laboratory of Luminescent Materials and Devices, Guangdong Provincial Key Laboratory of Luminescence from Molecular Aggregates, South China University of Technology 510640 Guangzhou China mszjzhao@scut.edu.cn; State Key Laboratory of Optoelectronic Materials and Technologies (Sun Yat-sen University), School of Chemistry, Sun Yat-sen University Guangzhou 510275 China; Instrumental Analysis & Research Center, Sun Yat-sen University Guangzhou 510275 China

## Abstract

A pair of axially chiral organic enantiomers were facilely prepared through a one-pot sequential synthesis. They exhibit circularly polarized luminescence activities and have thermally activated delayed fluorescence (TADF) and aggregation-induced emission enhancement properties. Meanwhile, these two enantiomers present remarkable and reversible thermochromism in the crystalline state, enabling dual-colour TADF switching between orange and red. However, when they form cocrystals, the resulting racemate shows opposite thermochromic behaviors. These intriguing results probably emanate from their different optical activities, leading to distinct molecular packing modes and molecular conformation variations. Moreover, information encryption based on thermochromism of organic enantiomers and their racemate has been presented for the first time. This work may expand the application scope of chiral organic luminogens and pave a new way to construct intelligent luminescent systems.

## Abstract 2

A pair of axially chiral organic enantiomers with circularly polarized thermally activated delayed fluorescence and aggregation-induced emission enhancement properties show opposite thermochromic behaviors to their racemate.

## Graphical abstract

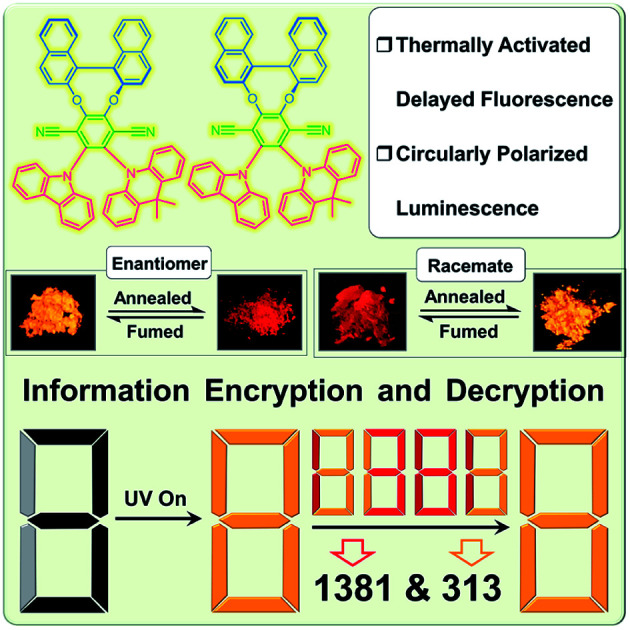

## Introduction

Chiral organic luminophores have received considerable attention recently due to their promising applications in optoelectronic devices, optical data storage, bioencoding, quantum computing, and asymmetric photochemical synthesis.^1–4^ In general, they can be constructed by introducing chiral luminescent center(s) or using the concept of chiral perturbation by tethering an enantiopure unit to a chromophore.^4–6^ Under the guidance of these design strategies, lots of chiral organic luminogens with unique luminescence properties such as thermally activated delayed fluorescence (TADF), room-temperature phosphorescence, and afterglow have been sequentially developed in the last few years.^2,4,7–14^ Despite these significant achievements, research on the differences in photophysical properties between chiral molecules and their racemate remains very limited.^15,16^ In principle, an emissive racemate and its enantiomers have identical chromophores. However, owing to the different optical activities, they inevitably form crystals with distinct space groups.^15^ As a result, their molecular packing modes are entirely different in the crystalline state. In most cases, the photophysical properties of organic compounds are closely related to their molecular arrangements.^17–20^ In this context, the enantiomers and their racemates can provide prototypes for studying the relationships among chemical structures, molecular packing modes, and luminescence performance of organic compounds, which are essential for understanding some peculiar photophysical processes and achieving new functional organic luminophores.^21,22^

Notably, purely organic materials with circularly polarized thermally activated delayed fluorescence (CP-TADF) have attracted great interest in recent years.^4,7,23–27^ They can harvest both singlet and triplet excitons and produce CP light directly, which are favorable for elevating the internal quantum efficiencies of organic light-emitting diodes (OLEDs) and diminishing the electroluminescence absorption loss on the polarizer to reduce energy consumption in OLED displays.^28–30^ However, probably due to the more complicated molecular design than common TADF emitters, only a few organic compounds with CP-TADF characteristics have been reported to date. In the meantime, it remains challenging to develop CP-TADF luminogens that can generate red light emission in the solid state.^1,2,4,31^ Moreover, the emission colours of CP-TADF emitters are usually tuned by molecular tailoring, requiring extra chemical syntheses and purification processes, which are tedious and time-consuming.^2,32^ Accordingly, it would be nice if their luminescence properties are switchable using a convenient approach, for instance, controlling the molecular packing mode *via* thermally annealing or fuming with solvent vapor. In addition, the CP-TADF luminogens and their racemate likely show different emission changes under the same external stimulus because of their different optical activities, which may be helpful for information encryption and anti-counterfeiting applications. Therefore, smart CP-TADF emitters, especially those that can produce red light in the solid state, are highly desirable to further unlock the tremendous technological potential of chiral organic luminophores.

In this article, we present a pair of CP-TADF active organic enantiomers and their racemate, which show different thermochromic behaviors, and demonstrate the possibility of their application in information encryption. Herein, carbazole and 9,9-dimethyl-9,10-dihydroacridine (DMAc) were selected as electron donors, and terephthalonitrile was employed as an electron acceptor to construct a luminescent molecule with a twisted donor–acceptor structure. Meanwhile, axially chiral 1,1′-binaphthalene was tethered to the terephthalonitrile unit for chiral perturbation. The resulting enantiomers exhibited not only CP-TADF characteristics but also aggregation-induced emission enhancement (AIEE) properties. Furthermore, they displayed remarkable and reversible thermochromism in the crystalline state, enabling dual-colour TADF switching between orange and red. However, their racemate underwent an opposite thermochromic process, in which its emission colour was converted from red to orange upon thermal annealing. In the light of these exciting results, a multiple data encryption–decryption model was successfully fabricated by utilizing the smart CP-TADF enantiomers and their racemate as emitters, demonstrating that they are promising for constructing intelligent luminescent systems.

## Results and discussion

The target enantiomers, *i.e.*, (*R*)-CzACN and (*S*)-CzACN, were facilely synthesized through a one-pot sequential procedure under alkaline conditions (Scheme S1†). After purification by column chromatography, their molecular structures were then characterized using nuclear magnetic resonance spectroscopy, high-resolution mass spectroscopy, elemental analysis, and single-crystal X-ray diffraction. The decomposition temperatures of (*R*)-CzACN and (*S*)-CzACN were determined to be 408 °C and 427 °C (Fig. S1†), respectively, suggesting that the compounds are highly thermally stable. UV-visible absorption spectra and photoluminescence (PL) spectra of the enantiomers in dilute cyclohexane solutions are shown in Fig. S2.† The absorption peaks in the range from 288 nm to 350 nm can be assigned to the π–π* transitions of carbazole, DMAc, binaphthalene, and the whole molecule. In comparison, the weak bands at around 422 and 493 nm are associated with the intramolecular charge transfer (ICT) transitions from carbazole and DMAc units to the terephthalonitrile moiety, respectively.^33^ The broad and structureless emission bands with a maximum at 555 nm probably originate from the radiative decays of the charge-transfer excited states because they strongly depend on solvent polarities. Specifically, the emission peaks of these two compounds shift to 612 nm in toluene and then to 705 nm in acetonitrile (Fig. S3†). Nevertheless, most longwave absorption bands and the excitation spectral profiles of the enantiomers are similar in varying solvents (Fig. S4 and S5†), indicating their solvent-polarity-independent ground state electronic structures and small dipole moments associated with the ICT transitions.^34^

**Fig. 1:**
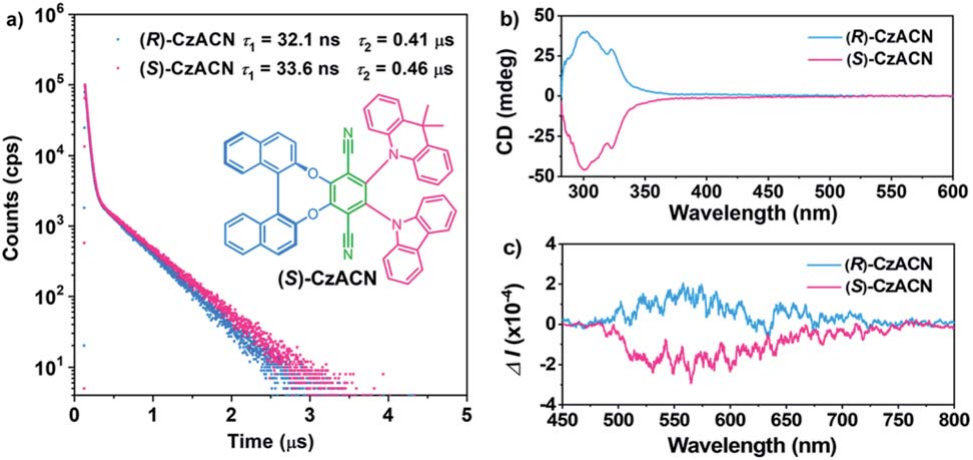
(a) Emission decay curves of the enantiomers in argon-degassed cyclohexane at room temperature. The inset shows the molecular structure of (*S*)-CzACN. (b) CD spectra of the enantiomers in toluene (50 μM). (c) CPL spectra of the enantiomers in cyclohexane under ambient conditions (0.2 mM).

Transient emission decays of (*R*)-CzACN and (*S*)-CzACN in solution were then measured at room temperature. As presented in Fig. 1a, the compounds emit distinctive delayed luminescence with long lifetimes of 0.41 μs and 0.46 μs, respectively, in addition to prompt fluorescence with typical short lifetimes of 32.1 ns and 33.6 ns in argon-degassed cyclohexane. However, in aerated solution, the lifetimes of the delayed components significantly reduce (Fig. S6†), suggesting that the delayed emissions arise from triplet excited states.^35^ Furthermore, the luminescence intensities of the enantiomers in cyclohexane under ambient conditions plummet in comparison with those in an inert atmosphere (Fig. S7†). Obviously, both (*R*)-CzACN and (*S*)-CzACN are TADF-active. Circular dichroism (CD) and circular polarized luminescence (CPL) spectroscopies were thereafter performed to investigate the chiroptical properties of the enantiomers in the ground and excited states, respectively. As shown in Fig. 1b, (*R*)-CzACN and (*S*)-CzACN show mirror-image bands in the CD spectra. The evident positive and negative Cotton effects of the enantiomers at around 288 nm, 300 nm, 311 nm, and 322 nm correspond to the characteristic π–π* transition absorption of carbazole, DMAc, and binaphthalene. Meanwhile, there are also weak Cotton effects from 400 nm to 500 nm, which can be assigned to the ICT transitions from the donors to the acceptor. As evidenced by these important signals, the chirality of binaphthalene is successfully transmitted to the achiral terephthalonitrile, carbazole, and DMAc moieties. Accordingly, the enantiomers are also capable of emitting distinct CPL. Fig. 1c shows the approximately symmetrical CPL spectra of (*R*)-CzACN and (*S*)-CzACN in cyclohexane solutions under ambient conditions. Their dissymmetric factor (*g*_lum_) were determined to be 3.02 × 10^−4^ and −3.12 × 10^−4^ (Fig. S8†), respectively, which are typical values for chiral small organic molecules. The clear CPL signals combined with the long lifetimes in microsecond order thus unambiguously demonstrate that these two enantiomers have CP-TADF characteristics. It is noteworthy that both (*R*)-CzACN (*Φ*_F,sl_ = 0.4%) and (*S*)-CzACN (*Φ*_F,sl_ = 0.4%) emit weak luminescence in dilute THF solutions. However, after 90% (v/v) distilled water was added, significant emission enhancements were recorded (Fig. S9†), and Mie scattering effects in the UV-visible absorption spectra were observed (Fig. S10†). In addition, nanoparticles with effective diameters of 376 nm and 329 nm were also detectable in the mixtures with 90% water fraction (Fig. S11†), indicating that the increase in the emission intensity of the compounds arose from the formation of nanoaggregates. These results signify that both (*R*)-CzACN and (*S*)-CzACN are CP-TADF luminogens with AIEE properties.^36,37^

**Fig. 2:**
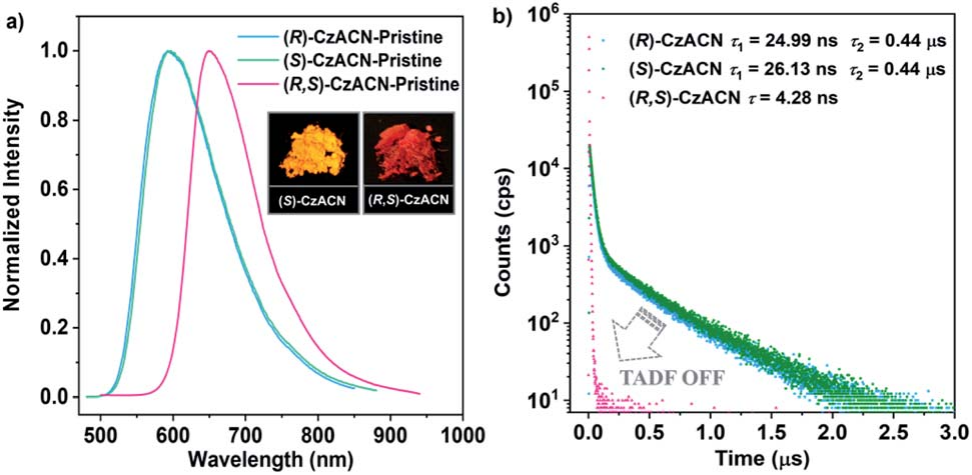
(a) PL spectra of the pristine samples of (*R*)-CzACN, (*S*)-CzACN, and (*R*,*S*)-CzACN. Insets show the luminescence images of the original microcrystals of (*S*)-CzACN and (*R*,*S*)-CzACN under the illumination of 365 nm UV light. (b) Emission decay curves of the pristine samples of (*R*)-CzACN, (*S*)-CzACN, and (*R*,*S*)-CzACN under ambient conditions.

Through reprecipitation from a mixture of dichloromethane (DCM)/methanol, these two enantiomers can form similar fibrous microcrystals. Under the excitation of UV light in air, the pristine microcrystals of (*R*)-CzACN (*Φ*_F,s_ = 3.9%) and (*S*)-CzACN (*Φ*_F,s_ = 5.3%) show identical orange fluorescence with a maximum at 592 nm (Fig. 2a). As expected, their luminescence significantly intensifies under vacuum, manifesting that the triplet excited states are involved in the luminescence processes (Fig. S12†). Emission decays of the enantiomers were then recorded (Fig. S13†), and the fitting gave a short lifetime and a long one for each of them (24.99 ns and 0.44 μs for (*R*)-CzACN and 26.13 ns and 0.44 μs for (*S*)-CzACN), suggesting that the chiral luminophores are also TADF active in the crystalline state.^38^ Their TADF characteristics are further validated by the temperature-dependent emission decay of the delayed components (Fig. S14†). Taking (*S*)-CzACN as an example, its lifetime increases from 1.21 μs at 77 K to 1.34 μs at 100 K and subsequently continuously decreases to 0.60 μs at 300 K.^39^ Similar results are also observed for (*R*)-CzACN. Intriguingly, when (*R*)-CzACN and (*S*)-CzACN form a racemate (hereafter denoted as (*R*,*S*)-CzACN), the resulting cocrystals exhibit a notable bathochromic shift in the emission maximum, giving red fluorescence centered at 645 nm (*Φ*_F,s_ = 1.2%). Moreover, both in air and under vacuum, no delayed component can be recorded in their emission decay curves (Fig. 2b and S15†), implying that the TADF from the chiral molecules is turned off. Therefore, the emission colours and the TADF attributes of the organic materials in the crystalline state can be facilely tuned by manipulating their optical activities. Given that the racemate and its enantiomers are bound to form different crystal structures, we speculate that such striking variations are likely associated with the alterations of molecular packing modes and molecular conformations.^40^

**Fig. 3:**
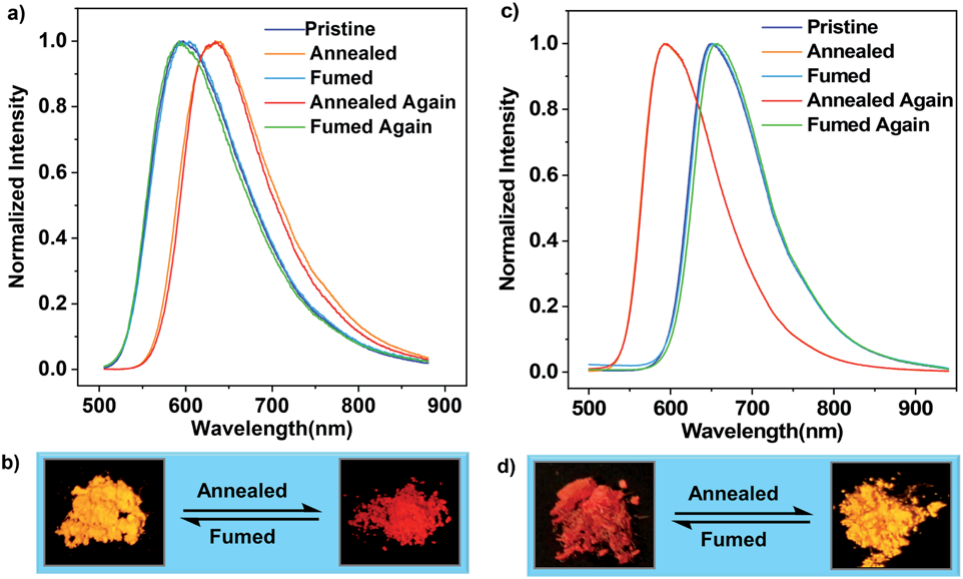
(a) Reversible thermochromism of (*S*)-CzACN. The annealed samples were obtained by annealing the pristine and fumed samples at 300 °C for 10 min, while the fumed samples were prepared by fuming the annealed samples in DCM vapor for about 15 min. (b) Luminescence images for the pristine and annealed samples of (*S*)-CzACN. (c) Reversible thermochromism of (*R*,*S*)-CzACN. The annealed samples were obtained by annealing the pristine and fumed samples at 260 °C for 5 min. The fumed samples were prepared by fuming the annealed samples in DCM vapor for about 5 min. (d) Luminescence images for the pristine and annealed samples of (*R*,*S*)-CzACN.

Differential scanning calorimetry (DSC) was conducted to investigate the morphological stabilities of the samples. It is found that three transitions exist in the first heating DSC thermograms of the pristine microcrystals of (*R*)-CzACN and (*S*)-CzACN (Fig. S16†). While the broad endothermic band in the range of 200 °C to 275 °C and the sharp exothermic peak at around 285 °C are probably related to the crystal structure transformation, the endothermic peak at 348 °C may represent the melting of the new ordered phase. To verify these hypotheses, thermally annealed samples were prepared, and their morphological and photophysical properties were characterized. Fig. S17† shows the distinct XRD patterns of the annealed powders compared with those of the pristine samples. Moreover, only a single endothermal peak that corresponds to melting is present in the DSC curves of the annealed powders. Collectively, the endothermic and exothermic transition signals prior to the melting peak in the DSC thermograms of pristine microcrystals originate from molecular rearrangements, which are likely associated with the destruction and reconstruction of intermolecular interactions, respectively. Notably, we have found that thermal annealing also triggers an alteration in the luminescence colour of the pristine microcrystals, from orange to red with an emission maximum at 636 nm (Fig. 3a and b, and S18†), enabling remarkable thermochromism for the enantiomers, which is seldom reported for CP-TADF emitters. Furthermore, the thermally annealed samples of (*R*)-CzACN (*Φ*_F,s_ = 3.3%) and (*S*)-CzACN (*Φ*_F,s_ = 3.5%) are likewise TADF-active, as indicated by the prompt and delayed components in their transient emission decay curves (Fig. S19†). More impressively, the red powders can entirely turn back to the orange ones upon fuming with DCM vapor for about 15 min. These experimental results are fully supported by the recoveries of the PL spectra, DSC curves, and XRD patterns. Accordingly, the enantiomers (*R*)-CzACN and (*S*)-CzACN with CPL and AIEE properties exhibit dual-colour TADF switching between orange and red after being heated and fumed, which may be applied to anti-counterfeiting and optoelectronic devices.

In the case of pristine (*R*,*S*)-CzACN, its DSC thermogram also presents a broad endothermic band in the range of 115 °C to 215 °C and a small exothermic peak at around 234 °C (Fig. S20a†), allowing the racemate to change its crystal structure *via* thermal treatment. Consequently, when the as-prepared cocrystals were heated at 260 °C for about 5 min, a new crystalline sample with a distinctly different molecular stacking mode could be obtained, which was identified by the variations of diffraction peaks in the XRD pattern (Fig. S20b†) and the disappearance of thermal transitions in the DSC curve. No melting peak can be observed for the racemate, suggesting that the (*R*)-CzACN and (*S*)-CzACN molecules within the annealed sample may build robust intermolecular interactions, making the cocrystals decompose before melting. PL measurements were subsequently carried out to survey the photophysical properties of the resulting crystals (Fig. 3c). Unexpectedly, thermally annealing the original sample of (*R*,*S*)-CzACN induced a substantial hypochromic shift in the emission peak (*λ*_max,em_ = 592 nm, *Φ*_F,s_ = 4.9%) and made the luminescence colour change from red to orange (Fig. 3d). The PL maximum of the annealed powder is in accordance with those of the pristine microcrystals of (*R*)-CzACN and (*S*)-CzACN, implicating that the chiral molecules within the orange crystals of (*R*,*S*)-CzACN and the enantiomers likely adopt similar molecular conformations. Evidently, the racemate exhibits opposite thermochromic behaviors to the enantiomers, which has not been reported before. The foregoing experimental results thus manifest that altering the optical activities of organic materials can not only tune their molecular packing and emission properties but also impact their luminescence behaviours under the same external stimulation. The thermally annealed sample of (*R*,*S*)-CzACN shows prompt (*τ* = 23.02 ns) and delayed (*τ* = 0.67 μs) components in its transient emission decay curve in air (Fig. S21†), suggesting its TADF characteristics. Such an inference is further certified by its longer lifetime and higher emission intensity under vacuum and increased delayed components with temperature elevation (Fig. S22†), that is, the thermal stimulation simultaneously varies the luminescence colour and turns on the TADF of the racemate. Similarly, by exposing the thermally annealed crystals to DCM vapor for about 5 min, the emission of the racemate can be fully restored. The recuperation of the emission colour upon repeated annealing–fuming cycles demonstrates that the thermochromism of the racemate is likewise reversible, which is also verified by the recoveries of the XRD patterns and DSC curves. Notably, the pristine and annealed samples of the enantiomers and racemate, which have been stored in air at room temperature for more than half a month, exhibit similar PL spectra and XRD patterns to the freshly prepared ones (Fig. S23–S26†), suggesting that their packing modes and the conformations of the involved chiral molecules are unchanged. These results fully demonstrate that the solids composed of the enantiomers and racemate build robust intermolecular interactions and have high thermodynamic stabilities.

Single crystal analysis and theoretical calculations were subsequently implemented to gain in-depth insight into the relationship among the optical activities, molecular packing, photophysical properties, and thermochromic behaviors of the enantiomers and their racemate. Through slow solvent evaporation, fibrous single crystals (SC_eo_) and lamellar single crystals (SC_er_) of (*S*)-CzACN, whose emission maxima are consistent with those of the pristine and annealed samples (Fig. S27†), were isolated from mixtures of DCM/ethanol and DCM/ethyl acetate, respectively. Concurrently, prismatic single crystals of (*R*,*S*)-CzACN (SC_rr_), with a PL spectrum that is almost identical to the one of its pristine sample, were also achieved from the mixture of DCM/ethanol by means of solvent evaporation. Although the orange-light-emitting single crystals of (*R*,*S*)-CzACN were not obtained, analysis of the SC_eo_ crystals of (*S*)-CzACN may provide useful information to decipher the thermochromic mechanism of the racemate. The annealed microcrystals of (*R*,*S*)-CzACN and the pristine microcrystals of (*S*)-CzACN show identical orange TADF, suggesting that the involved chiral molecules likely have similar molecular conformations.

**Fig. 4:**
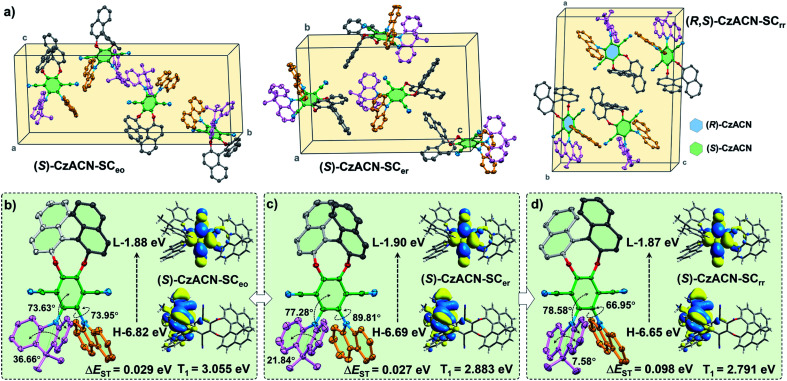
(a) Molecular packing modes of the chiral molecules in the SC_eo_ and SC_er_ single crystals of (*S*)-CzACN and the SC_rr_ single crystal of (*R*,*S*)-CzACN. (b)–(d) Molecular conformation of (*S*)-CzACN in the SC_eo_, SC_er_, and SC_rr_ single crystals, respectively. Insets show the Kohn–Sham frontier orbitals, Δ*E*_ST_ value, and T_1_ energy level of (*S*)-CzACN obtained from theoretical simulation. H and L represent the HOMO and LUMO, respectively.

In the SC_eo_ crystals, the enantiomer exhibits a half-slipped herringbone packing, and each unit cell contains four bulky molecules (Fig. 4a and S28a†). The dihedral angles between terephthalonitrile and DMAc (denoted as *θ*_1_) as well as carbazole (denoted as *θ*_2_) were measured to be 73.63° and 73.95°, respectively, while the bend angle of DMAc (denoted as *θ*_3_) was confirmed as 36.66° (Fig. 4b). Thus, the chiral molecules have highly twisted molecular conformations, indicating that no π–π stacking is observed among them. Meanwhile, lots of intermolecular interactions, including C–H⋯N and C–H⋯π, are found in the crystal structure (Fig. S28c†). These multiple interactions can immobilize the molecular conformations and hinder the intramolecular motions, which largely suppress the nonradiative decays, thereby enabling the enantiomers' AIEE effects. As indicated by the theoretical simulation results, the LUMO (*E* = −1.88 eV) of the molecule concentrates on terephthalonitrile, while the HOMO (*E* = −6.82 eV) mainly locates at DMAc and slightly extends to the carbazole unit. Accordingly, the luminescence properties of the enantiomers largely depend on the conjugation and charge transfer strength of these three chromophores. In particular, the DMAc substituent probably makes more contribution to the emissions of the enantiomers in comparison with carbazole.^25,33^

For the SC_er_ crystals, the containing (*S*)-CzACN molecules also exhibit a twisted molecular conformation and adopt an anti-parallel packing mode (Fig. S29†). Their DMAc moieties become much more planar (*θ*_3_ = 21.84°) in comparison with those of the molecules in the SC_eo_ crystals (Fig. 4c), which may dramatically increase the molecular conjugation and increase the HOMO energy level. On the other aspect, the *θ*_1_ and *θ*_2_ angels increase to 77.28° and 89.81°, respectively, probably enhancing the ICT strength and decreasing the LUMO energy level of the molecules. Such inferences are supported by the calculation results for the HOMO (*E* = −6.69 eV) and LUMO (*E* = −1.90 eV) of (*S*)-CzACN in the SC_er_ crystals and the fact that redshifts of the most longwave absorption bands were observed for the annealed samples of the enantiomers (Fig. S30†). The HOMO–LUMO energy gap (Δ*E*_g_) of the chiral molecule then reduces, leading to the significant bathochromic shift in the emission maximum for the SC_er_ crystals. As a result, the thermochromism of the pristine microcrystals of (*R*)-CzACN and (*S*)-CzACN likely originates from the change of molecular arrangement under thermal stimulation, which induces the planarization of the DMAc unit and the enlargement of the dihedral angles between the acceptor and the two donors. In addition, the singlet–triplet energy gaps (Δ*E*_ST_) of the chiral molecules in the SC_eo_ and SC_er_ crystals are calculated to be 0.029 eV and 0.027 eV, respectively. The small Δ*E*_ST_ values can facilitate the reverse intersystem crossing (rISC) processes in (*S*)-CzACN and then make the emitters produce orange and red TADF (Fig. S32†).

When (*R*)-CzACN and (*S*)-CzACN form cocrystals, they arrange alternately and stack layer by layer (Fig. S31†). In the unit cells of SC_rr_, both the optical isomers have two molecules, and their molecular conformations are identical except for the chirality of the binaphthalene moiety. Taking (*S*)-CzACN as an example, its *θ*_3_ angle further decreases to 7.58° (Fig. 4d), demonstrating an almost planar conformation for the DMAc moiety. Thus, the molecular conjugation of the enantiomer probably further increases, which results in a higher HOMO energy level. Although the *θ*_1_ angle slightly increases (78.58°), the *θ*_2_ one dramatically decreases to 66.95°. Thus, the ICT strength may be weakened, resulting in an elevation in the LUMO energy level. The overall effect is that the Δ*E*_g_ value reduces again, which can be confirmed by the theoretical calculation results. Consequently, the chiral molecules in the pristine cocrystals of the racemate show a redder emission and a larger simulated Δ*E*_ST_ value (0.098 eV). In principle, they also have potential to produce TADF because their Δ*E*_ST_ values are smaller than 0.20 eV and still in the optimum region (Fig. S33†).^41^ However, the experimental data unambiguously demonstrated that no delayed emission was observed from them. In the light of this, the unstable triplet excited states resulting from their lower energy levels and the relatively active molecular motions, which are supported by the reduction of the *Φ*_F,s_ value, may be responsible for the loss of TADF properties of the red cocrystals. Upon thermal annealing, the conformations of the acceptor and the donors of (*R*)-CzACN and (*S*)-CzACN probably transform into ones that are similar to the chiral molecules in the SC_eo_ crystals, thereby blue shifting the emission and turning on the TADF of the (*R*,*S*)-CzACN cocrystals. Obviously, the different photophysical properties and thermochromic behaviors between the pristine samples of the enantiomers and the racemate should be attributed to their different optical activities, which lead to the distinct molecular packing modes and conformation variations of the acceptor and the two donors.

**Fig. 5:**
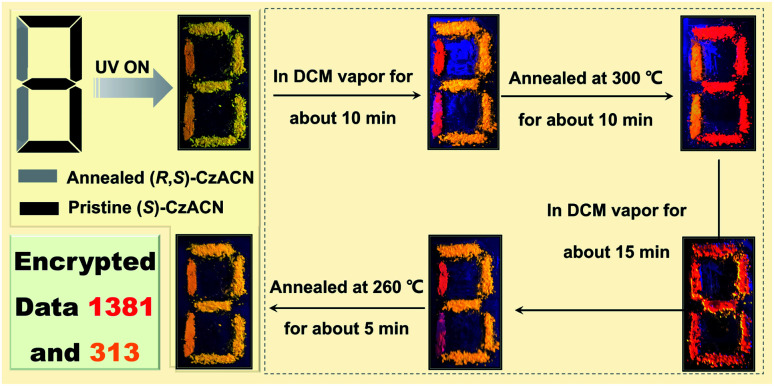
A multiple data encryption–decryption model fabricated by employing (*S*)-CzACN and (*R*,*S*)-CzACN as emitters. Two groups of encrypted numbers, *i.e.*, “1381” and “313”, can be deciphered through the red and orange channels by thermally annealing and fuming with solvent vapor, respectively.

Nowadays, information security has received significant attention from society.^10,42,43^ Here, a multiple data encryption–decryption model was fabricated by utilizing the reversible thermochromic properties of the racemate and the enantiomers. As depicted in Fig. 5, a digit pattern “8” was constructed on a glass slide, in which the annealed powder of (*R*,*S*)-CzACN and the pristine microcrystals of (*S*)-CzACN were employed as emitters to label the grey part and the black section, respectively. Under the illumination of 365 nm UV light, the pattern emitted orange TADF and displayed a digit “8” as the encryption state. By fuming with DCM vapor for about 10 min, the molecular arrangement of (*R*,*S*)-CzACN was altered, whereas the packing mode of (*S*)-CzACN remained unchanged. As a result, a red “1” and an orange “3” could be captured by the naked eye. When the resulting pattern was annealed at 300 °C for about 10 min, the thermochromic phenomena of (*R*,*S*)-CzACN and (*S*)-CzACN occurred simultaneously, decrypting an orange “1” and a red “3”, respectively. In fact, the luminescence of pristine (*R*,*S*)-CzACN and annealed (*S*)-CzACN looked quite similar because their emission maxima were very close (Δ*λ*_em_ = 9 nm). Moreover, the foregoing results demonstrated that the recovery time for the annealed sample of the racemate was shorter than those of the enantiomers. Thus, by fuming the annealed pattern with DCM vapor for about 15 min, a red digit “8” was further decoded, and subsequently, a red “1” and an orange “3” could be clearly observed. Eventually, two groups of encrypted numbers, *i.e.*, “1381” and “313”, were successfully deciphered by combining the red digits and the orange ones, respectively. More impressively, the data could be encrypted again *via* simply annealing the pattern at 260 °C for about 5 min, indicating that the encryption system has excellent secrecy performance, which is promising for practical applications in information security.

## Conclusions

In summary, for the first time, multiple data encryption based on thermochromism was successfully realized by controlling the luminescence of a pair of axially chiral organic enantiomers and their racemate. The enantiomers (*R*)-CzACN and (*S*)-CzACN present high thermal stabilities, as well as unique AIEE and TADF properties. They also exhibit obvious mirror-symmetric CD signals and desirable CPL activities. Moreover, these two enantiomers bear remarkable and reversible thermochromic characteristics, enabling dual-colour TADF switching between orange and red in the solid state. Unexpectedly, both (*R*)-CzACN and (*S*)-CzACN show opposite thermochromic behaviors to their racemate (*R*,*S*)-CzACN. Such intriguing photophysical phenomena probably result from their different optical activities, leading to the distinct molecular packing modes and conformation variations of the acceptor and the two donors. Inspired by the distinctive dual-colour TADF switching properties, a simple information encryption model with excellent secrecy performance has been fabricated by employing (*S*)-CzACN and (*R*,*S*)-CzACN as emitters. All these impressive results fully demonstrate that the enantiomers and their racemate hold promising potential for data encryption, anti-counterfeiting, sensing, and display.

## Data availability

The experimental details supporting this article are available in the ESI.† The crystallographic information files can be found with the supplemental materials associated with this work, and on the Cambridge Crystallographic Data Center under deposition numbers 1846015, 2092971 and 2092972.†

## Author contributions

B. Xu, G. Shi, and Z. Zhao designed the experiments. B. Xu, Z. Song, M. Zhang, Q. Zhang, L. Jiang, C. Xu, L. Zhong, C. Su and Q. Ban performed the experiments. B. Xu wrote the manuscript. All the authors were involved in the analysis and interpretation of the data.

## Conflicts of interest

There are no conflicts to declare.

## Supplementary Material

SC-012-D1SC04738H-s001

SC-012-D1SC04738H-s002
