# Supplementary material for: Controlling the thermally activated delayed fluorescence of axially chiral organic emitters and their racemate for information encryption
Source: Chem Sci. 2021 Nov 18;12(47):15556–62. doi: 10.1039/d1sc04738h (PMC8653998; doi:10.1039/d1sc04738h)
Supplement: SC-012-D1SC04738H-s001 [file SC-012-D1SC04738H-s001.pdf]

Electronic Supplementary Material (ESI) for Chemical Science.
This journal is © The Royal Society of Chemistry 2021

Controlling the Thermally Activated Delayed Fluorescence of Axially Chiral Organic Emitters and Their Racemate for Information Encryption

Bingjia Xu,* Zicun Song, Minmin Zhang, Qingqing Zhang, Long Jiang, Cao Xu, Lijun Zhong, Changlin Su, Qiqi Ban, Cong Liu, Fengqiang Sun, Yi Zhang, Zhenguo Chi, Zujin Zhao,* Guang Shi*

Content

1 Experimental Section 2
1.1 Chemical Reagents and Materials 2
1.2 Measurements 2
1.3 Synthesis 3
2 Thermal Stability 5
3 Photophysical Properties and TADF Characteristics 5
4 Dissymmetric Factors of the Enantiomers 7
5 Aggregation-Induced Emission Enhancement 8
6 TADF Activities and Thermochromism Properties in the Solid State 9
7 Single Crystal Structure Analysis and Luminescence Mechanism 14
8 ^{1}H NMR Spectra, ^{13}C NMR Spectra, and Mass Spectra 18
9 Data for the Single Crystal Structures 22
10 References 22

1 Experimental Section

1.1 Chemical Reagents and Materials

2,3,5,6-Tetrafluoroterephthalonitrile, (*R*)-(-)-1,1'-Bi-2-naphthol, (*S*)-(+)-1,1'-Bi-2-naphthol, carbazole, and super dry *N*,*N*-dimethylformamide were purchased from *J&K* Scientific. 9,9-Dimethyl-9,10-dihydroacridine was purchased from Suzhou Geao Company (China). All the materials noted above were used as received. Other solvents and reagents were purchased from Guangzhou Zeyuan Company (China) with analytical grade and used without further purification.

1.2 Measurements

Nuclear magnetic resonance (^{1}H NMR and ^{13}C NMR) spectra were measured on a Bruker AVANCE 400 MHz spectrometer (in $CDCl_3$ with internal standard of tetramethylsilane). Mass spectra were conducted on DSQ & MAT95XP-HRMS thermospectrometers. Effective diameters of the nanoaggregates in H_2O/THF mixtures with 90% water fraction were investigated on a Malvern Zetasizer (Nano ZS90). Thermal transitions of the samples were determined by differential scanning calorimetry (DSC) at a heating rate of 10 ºC/min under N_2 atmosphere using a TA thermal analyzer (Q20). Wide-angle XRD patterns were achieved at 293 K by a Bruker X-ray diffractometer (D8 ADVANCE, Germany) in a scan rate of 4° (2θ)/min. PL spectra were obtained on an Ocean Optics spectrophotometer (QE65 Pro) with a 365 nm LLS-LED as excitation source. UV-visible absorption spectra were monitored on a Hitachi U-3900H spectrophotometer. CPL spectra were measured on a JASCO CPL-300 spectrophotometer. Circular dichroism (CD) spectra were characterized by using a CD spectrometer (J-815) from JASCO instrument. Time-resolved emission decay curves and absolute PL quantum yields (PLQYs) were recorded on a spectrometer (FLS980) equipped with a calibrated integrating sphere from Edinburgh Instruments.

Single-crystal X-ray diffraction data for the enantiomers and racemate was obtained from an Agilent Technologies Gemini A Ultra system with Cu-Kα radiation (λ = 1.54178 Å). The structures were solved using direct methods following the difference Fourier syntheses. All non-hydrogen atoms were anisotropically refined through least-squares on F^2 using the Olex2 program suite. CCDC 1846015, 2092971 and 2092972 contain the supplementary crystallographic data for this paper. Theoretical calculations based on time-dependent density functional theory (TD-DFT) were

conducted on Gaussian 16 program with a method similar to previous literature.[1] Ground state (S_0) geometries of (*S*)-CzACN were directly selected from single crystal structures and were used as molecular models without further optimization. On the basis of this, Kohn–Sham frontier orbitals and energy levels of singlet (S_1) and triplet (T_1) excited states were simulated by using TD-DFT at the B3LYP/6-311G(p, d) level.

1.3 Synthesis

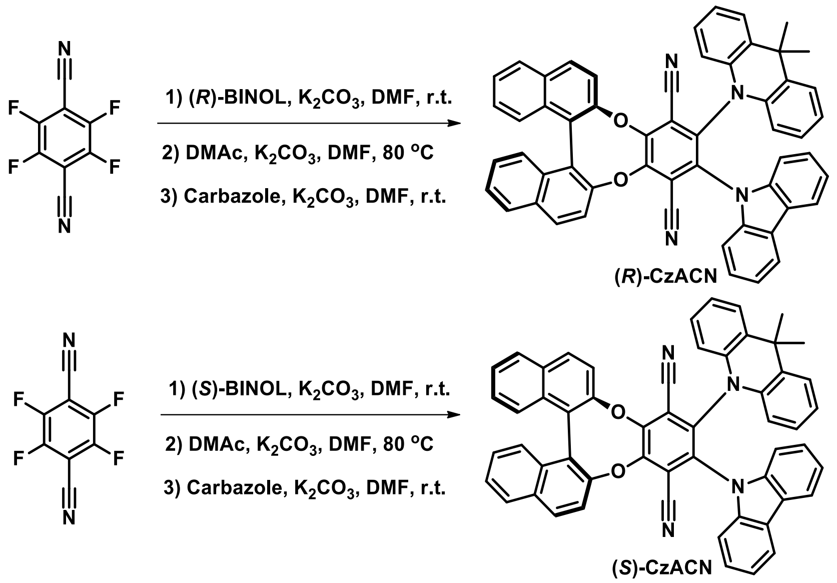

Scheme S1. Synthetic routes of the target compounds.

General procedures for the syntheses of target compounds

To a solution of 2,3,5,6-tetrafluoroterephthalonitrile (0.20 g, 1.00 mmol) and (*R*)-(-)-1,1'-bi-2-naphthol (or (*S*)-(+)-1,1'-bi-2-naphthol) (0.29 g, 1.00 mmol) in dry *N*,*N*-dimethylformamide (DMF) (12 ml) potassium carbonate (0.28 g, 2.00 mmol) was added. After the solution was stirred at room temperature under an argon atmosphere for 12 h, 9,9-dimethyl-9,10-dihydroacridine (0.21 g, 1.00 mmol) and potassium carbonate (0.14 g, 1.00 mmol) were added, and then the mixture was stirred at 80 °C for an additional 12 h. After cooling, carbazole (0.21 g, 1.25 mmol) and potassium carbonate (0.17 g, 1.25 mmol) were added, and the mixture was then stirred at room temperature for 6 h. The resulting mixture was poured to 200 ml of ice water, and the precipitate was collected and washed with water for several times. The crude product was purified by silica gel column chromatography with dichloromethane/petroleum ether/acetonitrile (v/v/v=10:60:2) as eluent.

***Synthesis of* (*R*)-CzACN** Compound (*R*)-CzACN was further precipitated from dichloromethane/ethanol under the action of ultrasonic to produce an orange powder in 39% yield (0.30 g). ^{1}H NMR

(400 MHz, $CDCl_3$): δ 8.23-8.12 (dd, *J*=13.7, 8.9 Hz, 2 H); 8.09-8.04 (dd, *J*=8.1, 4.0 Hz, 2 H); 7.89-7.82 (m, 2 H); 7.81-7.76 (d, *J*=8.8 Hz, 1 H); 7.75-7.71 (d, *J*=8.8 Hz, 1 H), 7.64-7.59 (m, 2 H), 7.57-7.53 (d, *J*=8.3 Hz, 2 H), 7.50-7.45 (m, 2 H), 7.18-7.15 (d, *J*=6.4 Hz, 1 H), 7.12-7.05 (m, 4 H), 7.02-6.97 (m, 2 H), 6.95-6.85 (m, 2 H), 6.85-6.77 (m, 3 H), 6.49-6.44 (d, *J*=8.2 Hz, 1 H), 6.27-6.22 (d, *J*=8.1 Hz, 1 H), 1.59-1.54 (s, 3 H), 0.14-0.07 (s, 3 H). ^{13}C NMR (101 MHz, DMSO-d_6): δ 153.07, 152.18, 149.36, 149.33, 140.39, 140.36, 139.56, 138.73, 138.55, 137.74, 132.48, 132.45, 131.74, 130.39, 130.31, 129.20, 128.26, 127.10, 127.07, 126.82, 126.58, 126.44, 125.82, 125.75, 125.30, 125.17, 124.52, 124.45, 123.41, 123.19, 122.28, 122.23, 121.80, 121.60, 120.85, 120.81, 120.41, 120.34, 117.84, 116.41, 114.58, 114.38, 112.83, 112.33, 111.31, 111.06, 35.37, 32.54, 26.26. EI-MS m/z: $[M]^+$ calcd for $C_{55}H_{34}N_4O_2$, 782; found, 782. HRMS m/z: $[M]^+$ calcd for $C_{55}H_{34}N_4O_2$, 782.2682; found, 782.2671. Anal. calcd for $C_{55}H_{34}N_4O_2$: C 84.38, H 4.38, N 7.16; found: C 83.74, H 4.13, N 6.93.

***Synthesis of* (*S*)-CzACN** Compound (*S*)-CzACN was further precipitated from dichloromethane/ ethanol under the action of ultrasonic to produce an orange powder in 36% yield (0.28 g). ^{1}H NMR (400 MHz, $CDCl_3$): δ 8.22-8.15 (dd, *J*=13.7, 8.9 Hz, 2 H); 8.09-8.04 (dd, *J*=8.2, 4.1 Hz, 2 H); 7.88-7.82 (m, 2 H); 7.80-7.76 (d, *J*=8.8 Hz, 1 H); 7.74-7.70 (d, *J*=8.8 Hz, 1 H), 7.63-7.59 (m, 2 H), 7.56-7.52 (d, *J*=8.4 Hz, 2 H), 7.50-7.44 (m, 2 H), 7.17-7.14 (d, *J*=7.7 Hz, 1 H), 7.11-7.04 (m, 4 H), 7.02-6.97 (m, 2 H), 6.94-6.85 (m, 2 H), 6.84-6.76 (m, 3 H), 6.49-6.44 (d, *J*=8.2 Hz, 1 H), 6.27-6.22 (d, *J*=8.1 Hz, 1 H), 1.58-1.54 (s, 3 H), 0.14-0.07 (s, 3 H). ^{13}C NMR (101 MHz, DMSO-d_6): δ 153.06, 152.18, 149.36, 149.33, 140.39, 140.36, 139.56, 138.73, 138.55, 137.74, 132.48, 132.45, 131.74, 130.39, 130.31, 129.20, 128.27, 127.10, 127.07, 126.82, 126.58, 126.44, 125.82, 125.76, 125.29, 125.17, 124.53, 124.44, 123.41, 123.19, 122.28, 122.23, 121.80, 121.60, 120.85, 120.81, 120.41, 120.34, 117.84, 116.41, 114.58, 114.38, 112.83, 112.32, 111.30, 111.07, 35.37, 32.54, 26.25. EI-MS m/z: $[M]^+$ calcd for $C_{55}H_{34}N_4O_2$, 782; found, 782. HRMS m/z: $[M]^+$ calcd for $C_{55}H_{34}N_4O_2$, 782.2682; found, 782.2671. Anal. calcd for $C_{55}H_{34}N_4O_2$: C 84.38, H 4.38, N 7.16; found: C 83.68, H 4.20, N 6.96.

***Preparation of* (*R,S*)-CzACN** Ethanol was added into a concentrated dichloromethane solution that contains (*R*)-CzACN and (*S*)-CzACN with molar ratio of 1:1 under the action of ultrasonic. Subsequently, the resulting precipitate was collected and dried to give (*R*,*S*)-CzACN as a red powder.

2 Thermal Stability

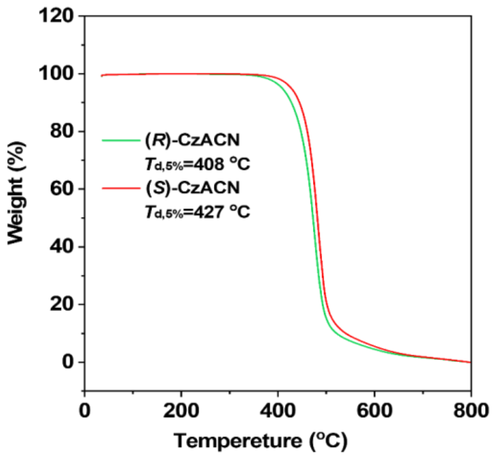

Fig. S1 TGA curves of (*R*)-CzACN and (*S*)-CzACN.

3 Photophysical Properties and TADF Characteristics

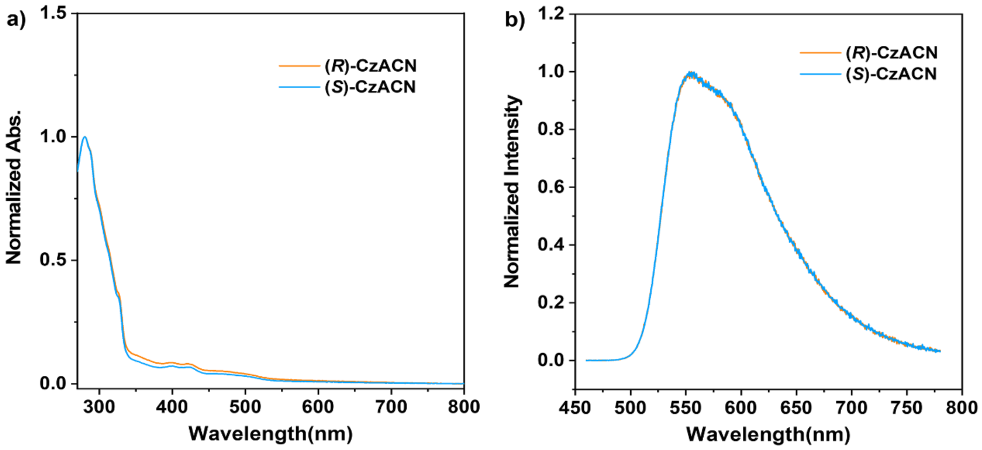

Fig. S2 UV-visible absorption a) and PL b) spectra of (*R*)-CzACN and (*S*)-CzACN in cyclohexane. (Concentration: 10 μM; Excitation: 330 nm)

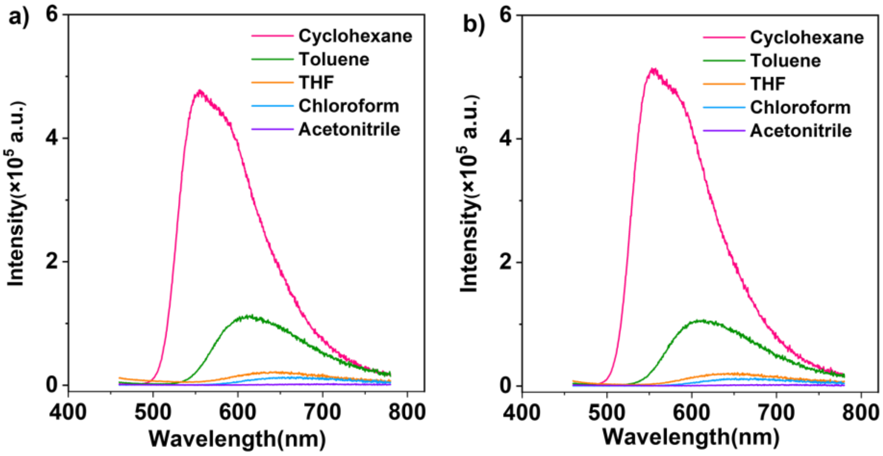

Fig. S3 PL spectra of (*R*)-CzACN a) and (*S*)-CzACN b) in different solvents. (Concentration: 10 μM; Excitation: 330 nm)

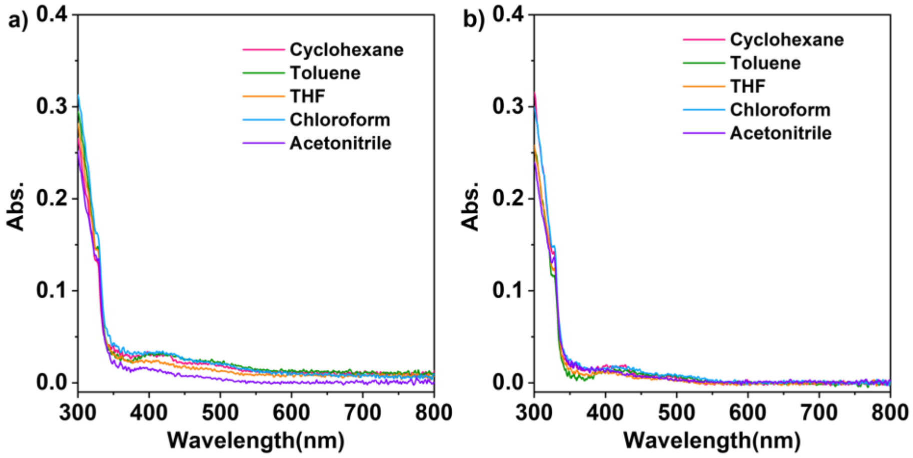

Fig. S4 UV-visible absorption spectra of (*R*)-CzACN a) and (*S*)-CzACN b) in different solvents. (Concentration: 10 μM)

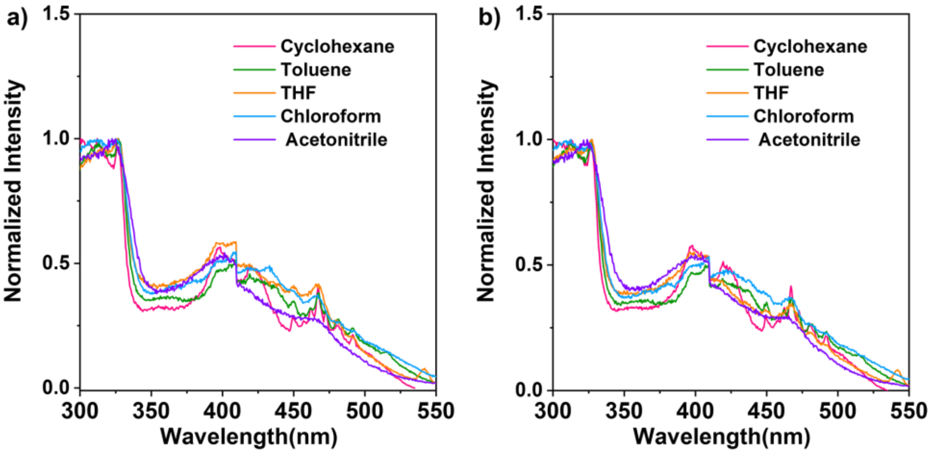

Fig. S5 Excitation spectra of (*R*)-CzACN a) and (*S*)-CzACN b) in different solvents. (Concentration: 10 μM)

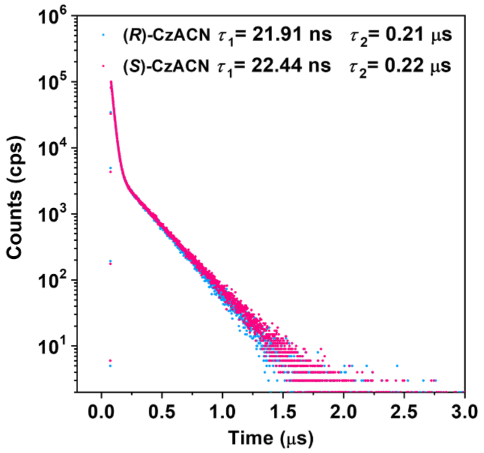

Fig. S6 Emission decay curves of the enantiomers in aerated cyclohexane at room temperature.

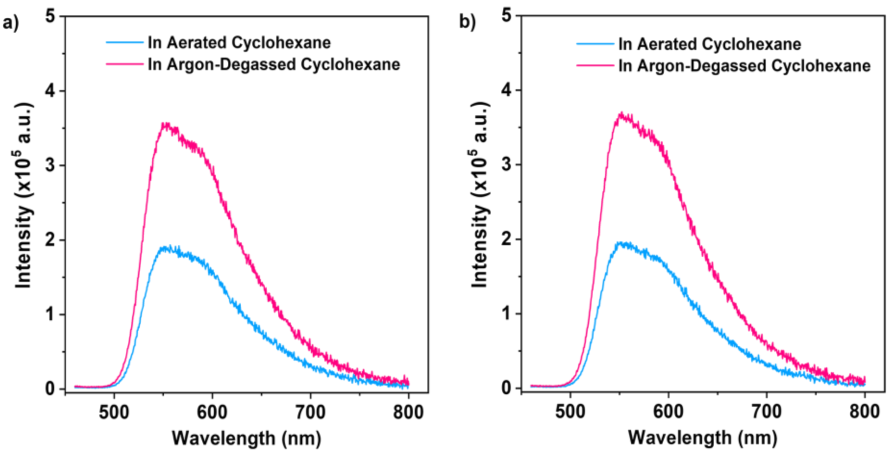

Fig. S7 PL spectra of (*R*)-CzACN a) and (*S*)-CzACN b) in cyclohexane under different atmospheres. (Concentration: 10 μM).

4 Dissymmetric Factors of the Enantiomers

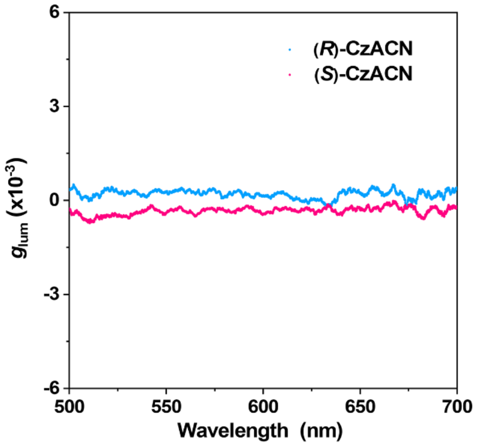

Fig. S8 Dissymmetric factors of (*R*)-CzACN and (*S*)-CzACN in cyclohexane at ambient condition. (Concentration: 0.2 mM)

5 Aggregation-Induced Emission Enhancement

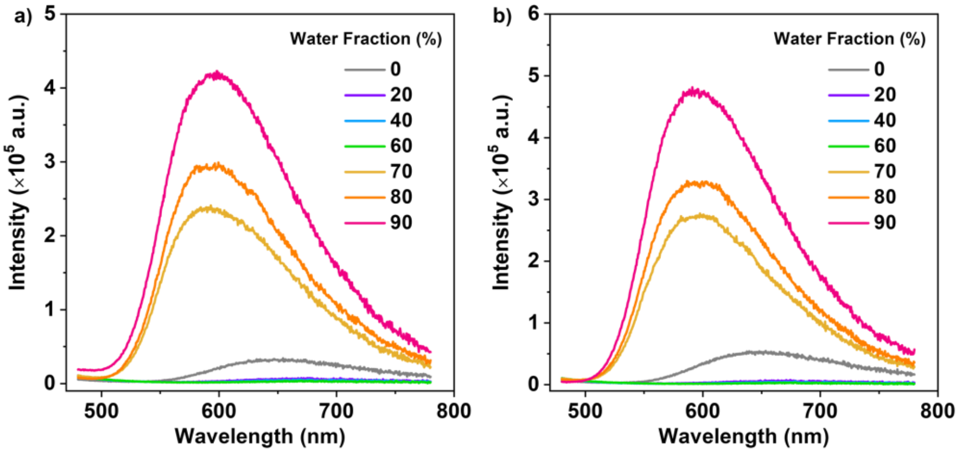

Fig. S9 PL spectra of the dilute solutions of (*R*)-CzACN a) and (*S*)-CzACN b) in water/THF mixtures with different water fractions. (Concentration: 10 μM)

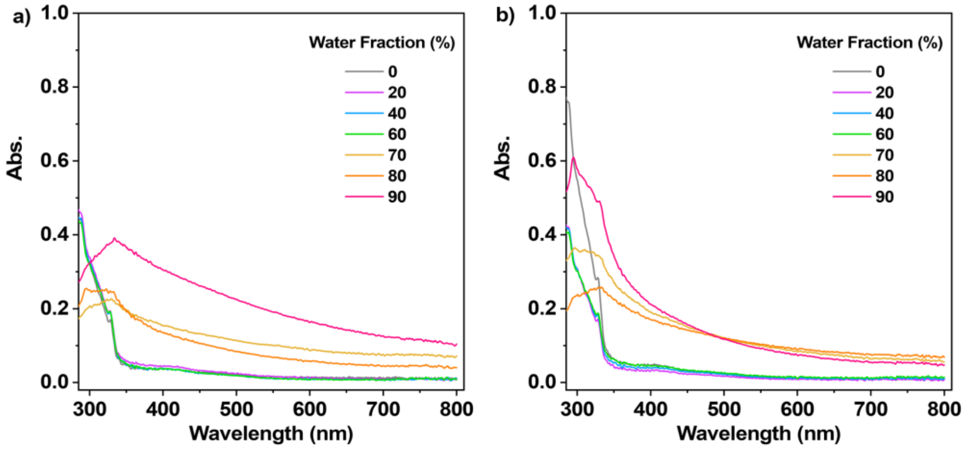

Fig. S10 UV-visible absorption spectra of the dilute solutions of (*R*)-CzACN a) and (*S*)- CzACN b) in water/THF mixtures with different water fractions. (Concentration: 10 μM)

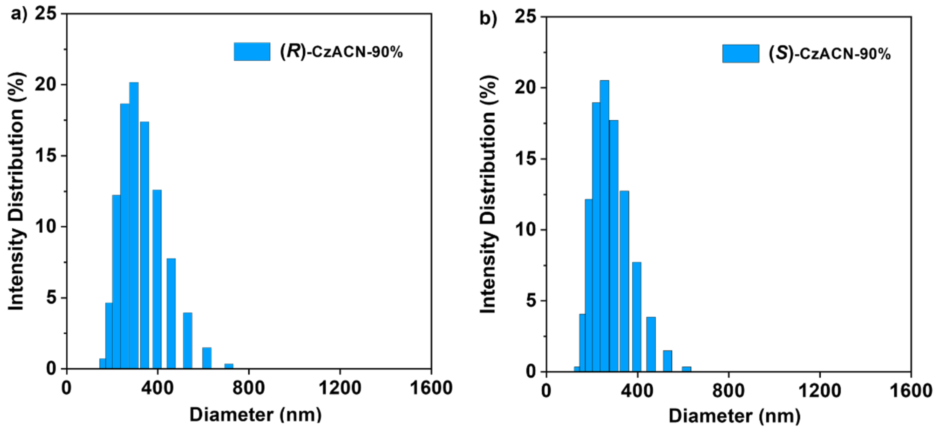

Fig. S11 Size distribution of the nanoparticles of (*R*)-CzACN a) and (*S*)-CzACN b) in water/THF mixtures with 90% water fractions. (Concentration: 10 μM)

6 TADF Activities and Thermochromism Properties in the Solid State

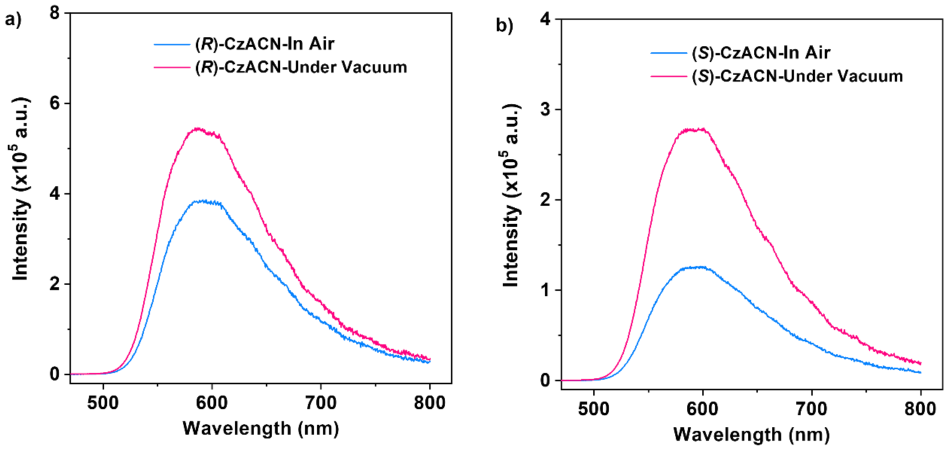

Fig. S12 PL spectra of the pristine samples of (*R*)-CzACN a) and (*S*)-CzACN b) under different atmospheres at room temperature.

After precipitation from a mixture of dichloromethane/methanol, the resulting microcrystals of (*R*)-CzACN and (*S*)-CzACN may contain trace of solvent molecules. Consequently, their weaker luminescence in air may be associated with the involved solvent molecules that dissolve oxygen, acting as a triplet quencher inside the microcrystals.

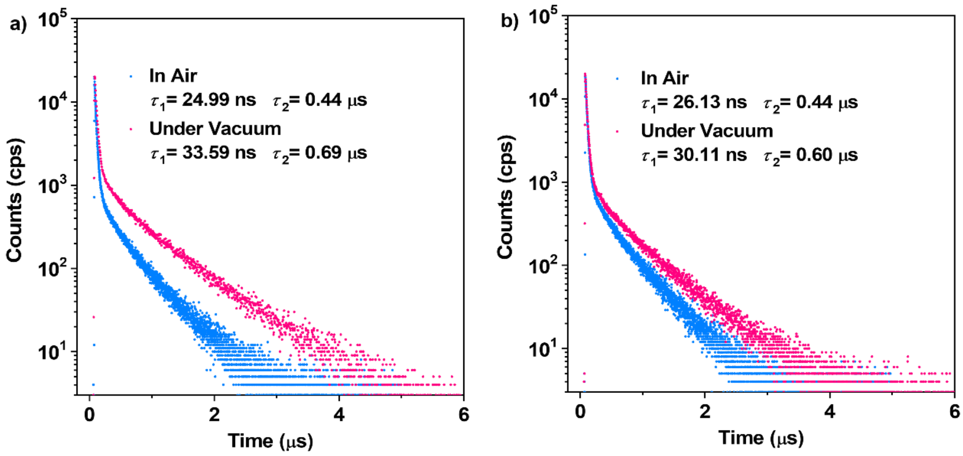

Fig. S13 Emission decay curves of the pristine samples of (*R*)-CzACN a) and (*S*)-CzACN b) under different atmospheres at room temperature.

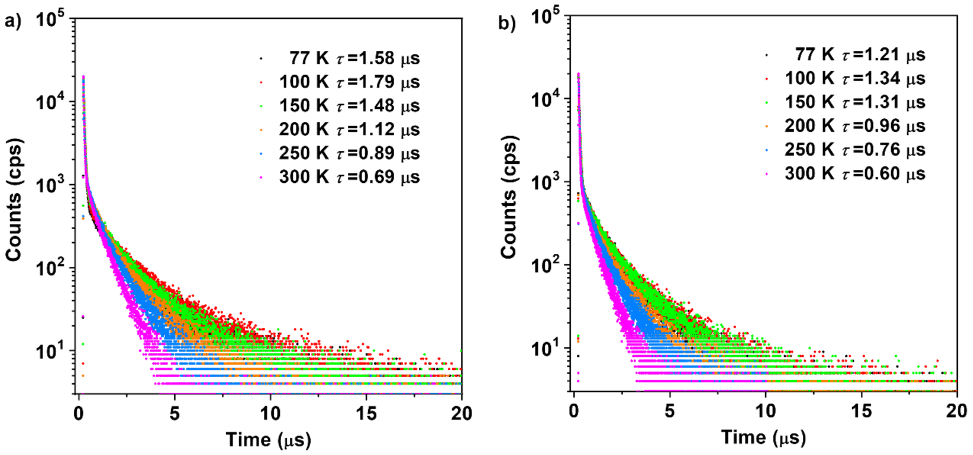

Fig. S14 Emission decay curves of the pristine samples of (*R*)-CzACN a) and (*S*)-CzACN b) under vacuum at different temperatures.

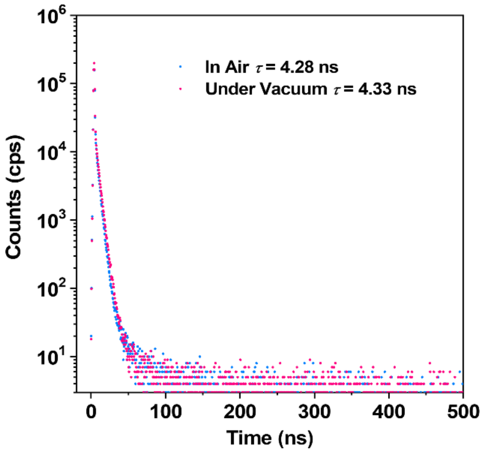

Fig. S15 Emission decay curves of the pristine sample of (*R*,*S*)-CzACN under different atmospheres at room temperature.

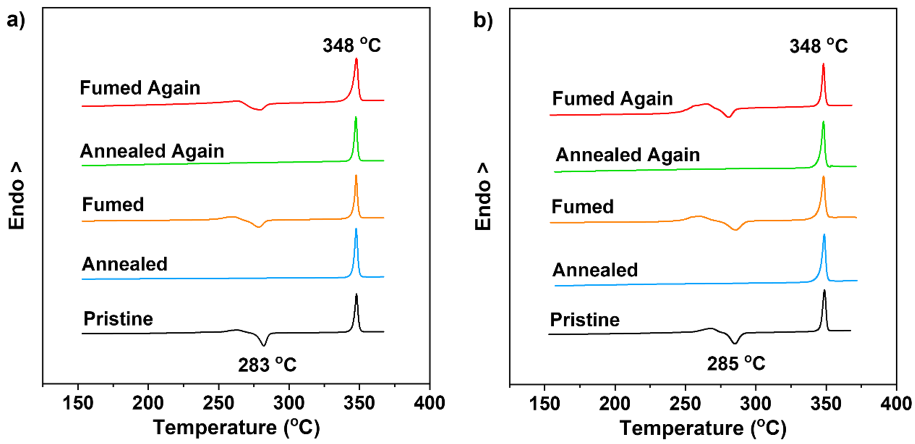

Fig. S16 DSC curves of the pristine, annealed, and fumed samples of (*R*)-CzACN a) and (*S*)-CzACN b).

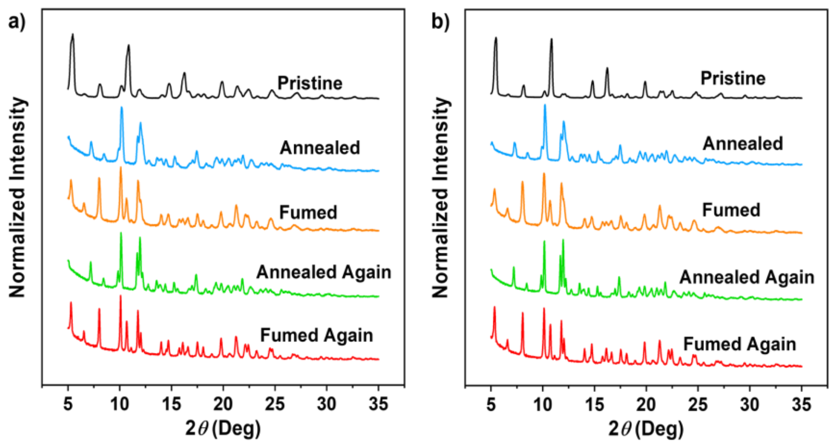

Fig. S17 XRD patterns of the pristine, annealed, and fumed samples of (*R*)-CzACN a) and (*S*)-CzACN b).

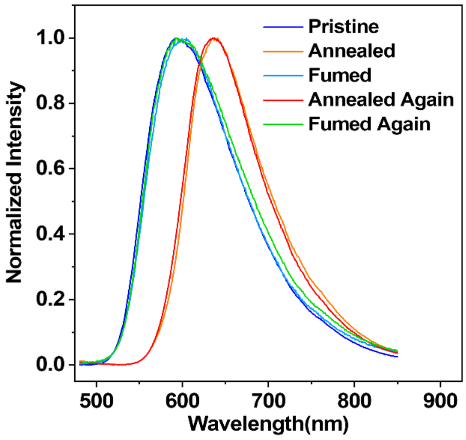

Fig. S18 Reversible thermochromism of (*R*)-CzACN. The annealed samples were obtained by annealing the pristine and fumed samples at 300 °C for 10 min, while the fumed samples were prepared by fuming the annealed samples in DCM vapor for about 15 min.

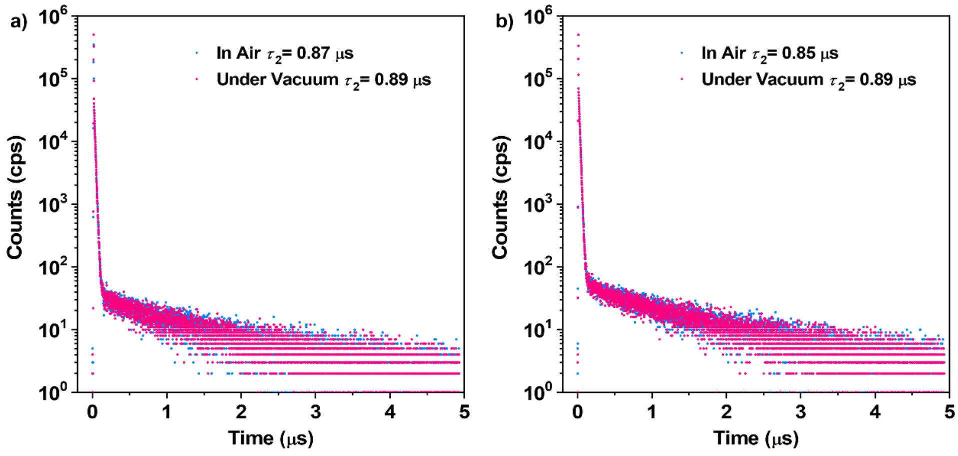

Fig. S19 Emission decay curves of the annealed samples of (*R*)-CzACN a) and (*S*)-CzACN b) under different atmospheres at room temperature.

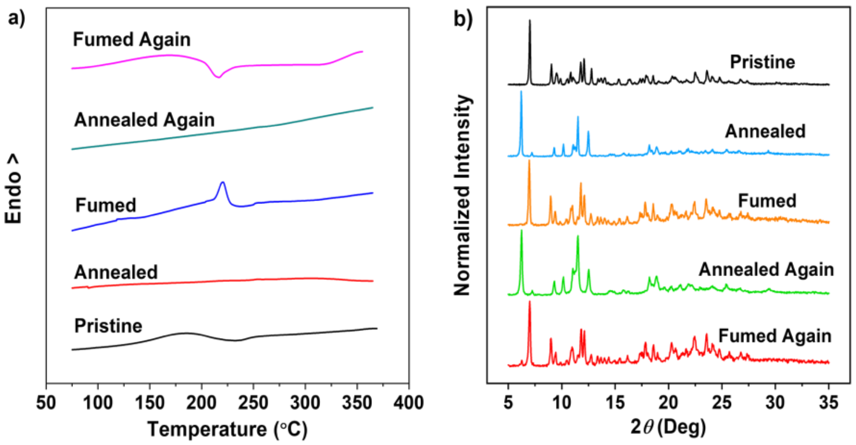

Fig. S20 a) DSC curves of the pristine, annealed, and fumed samples of (*R*,*S*)-CzACN. b) XRD patterns of the pristine, annealed, and fumed samples of (*R*,*S*)-CzACN.

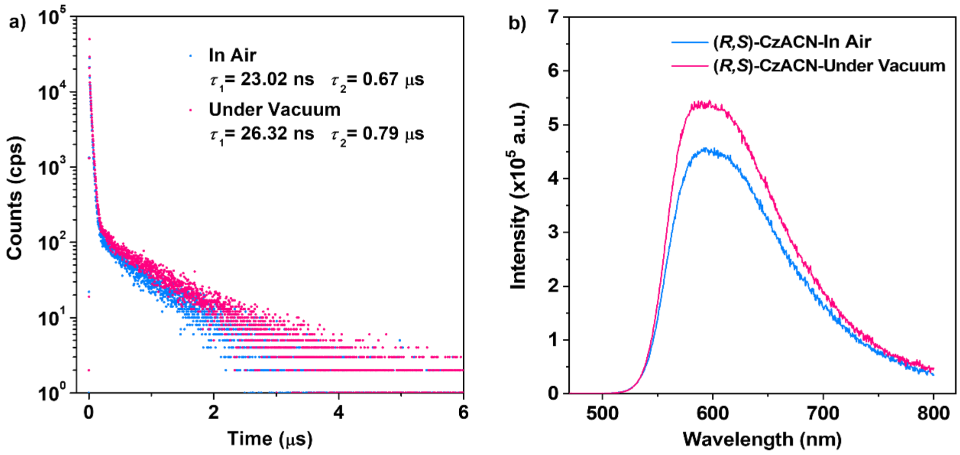

Fig. S21 Emission decay curves a) and PL spectra b) of the annealed sample of (*R*,*S*)-CzACN under different atmospheres at room temperature.

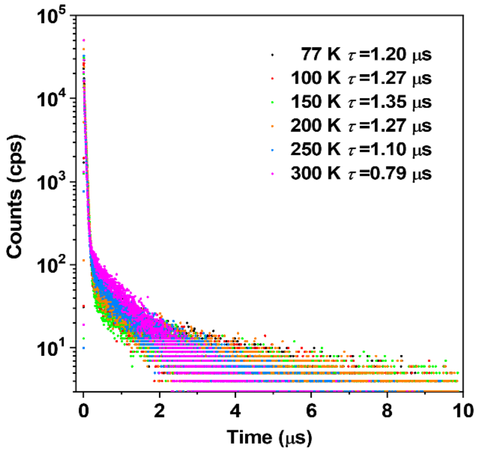

Fig. S22 Emission decay curves of the annealed sample of (*R*,*S*)-CzACN under vacuum at different temperatures.

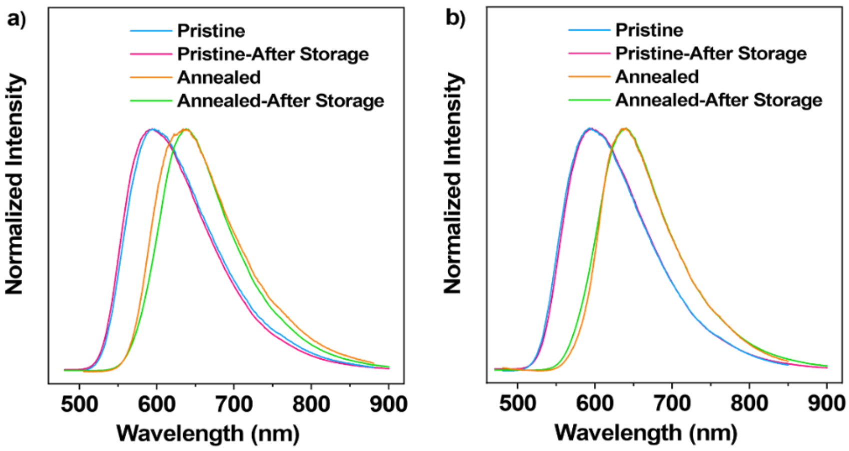

Fig. S23 PL spectra of the pristine and annealed samples of (*R*)-CzACN a) and (*S*)-CzACN b) before and after storage. The samples were stored in air at room temperature for more than half a year.

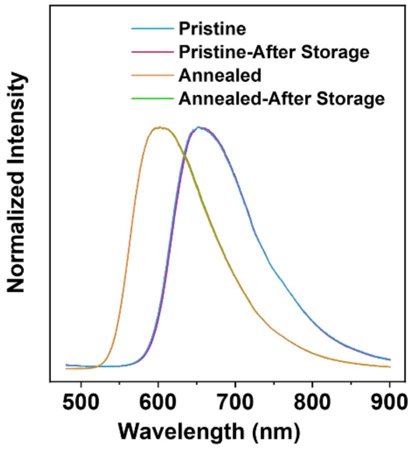

Fig. S24 PL spectra of the pristine and annealed samples of (*R*,*S*)-CzACN before and after storage. The samples were stored in air at room temperature for more than two weeks.

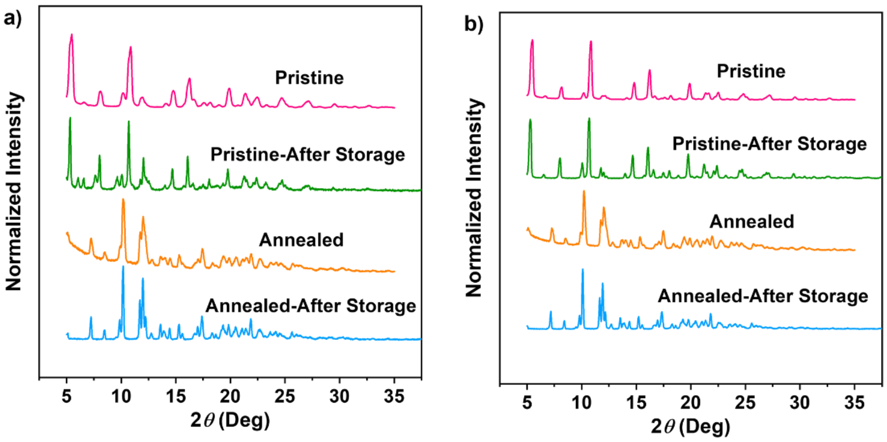

Fig. S25 XRD patterns of the pristine and annealed samples of (*R*)-CzACN a) and (*S*)-CzACN b) before and after storage. The samples were stored in air at room temperature for more than half a year.

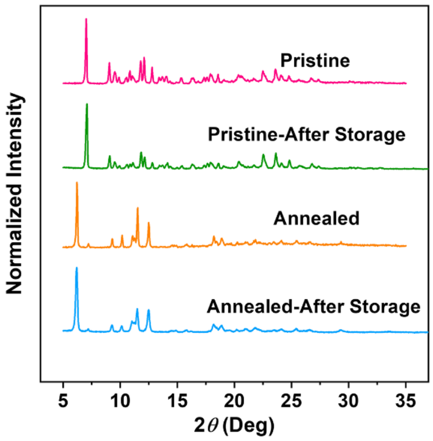

Fig. S26 XRD patterns of the pristine and annealed samples of (*R*,*S*)-CzACN before and after storage. The samples were stored in air at room temperature for more than two weeks.

7 Single Crystal Structure Analysis and Luminescence Mechanism

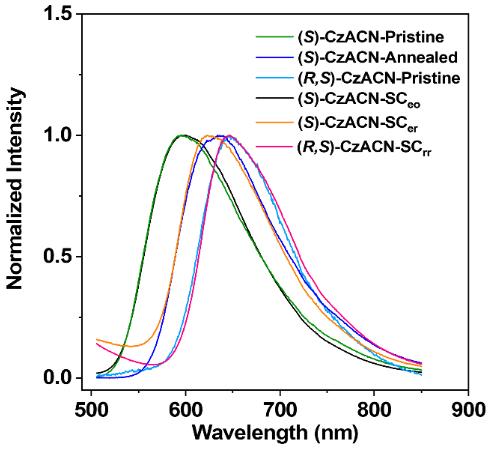

Fig. S27 PL spectra of the pristine samples, annealed samples, and single crystals of (*S*)-CzACN and (*R*,*S*)-CzACN.

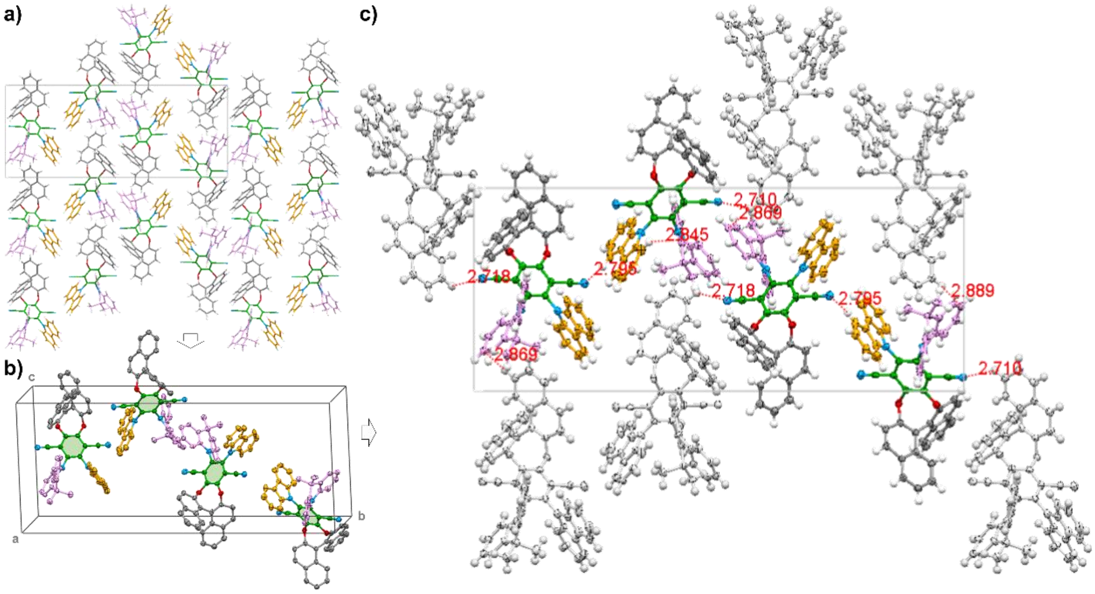

Fig. S28 Molecular arrangement and intermolecular interactions for the SC_{eo} single crystal.

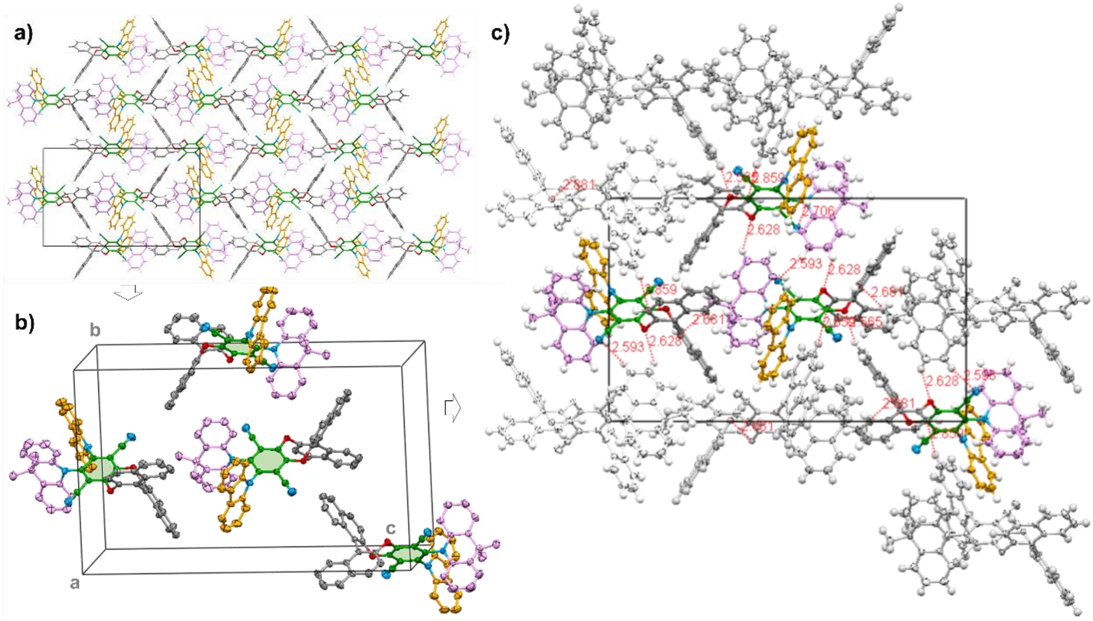

Fig. 29 Molecular arrangement and intermolecular interactions for the SC_{er} single crystal.

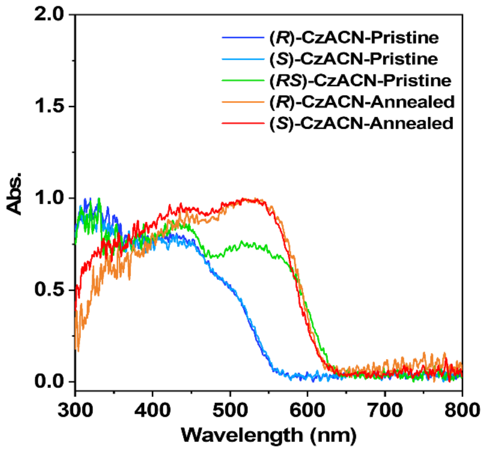

Fig. 30 UV-visible absorption spectra of the pristine and annealed samples.

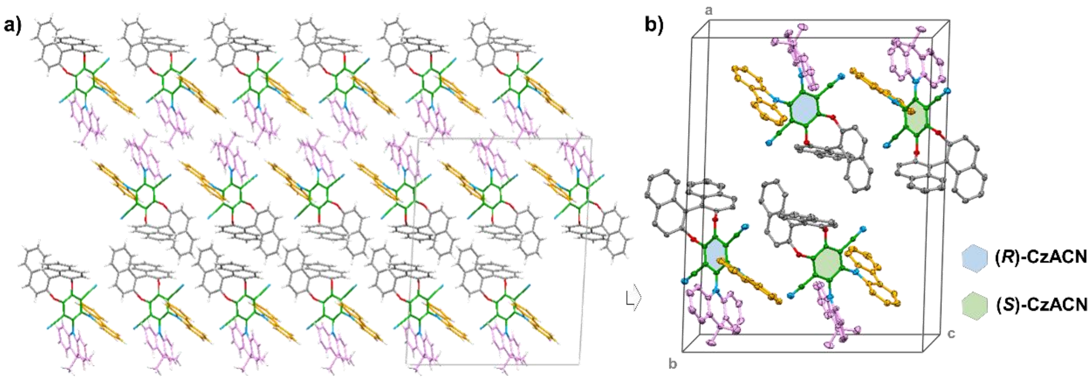

Fig. 31 Molecular packing mode and molecular arrangement for the SC_{rr} single crystal.

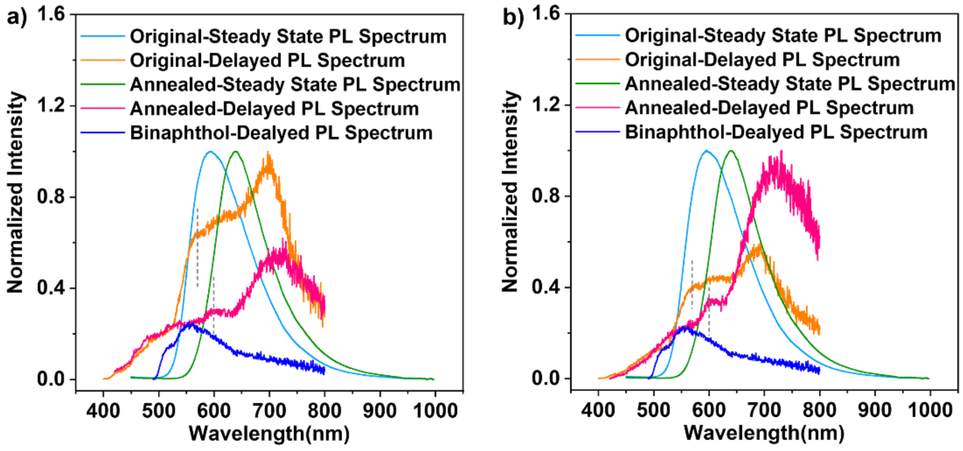

Fig. 32 Steady-state and delayed PL spectra for the pristine and annealed samples of (*R*)-CzACN a) and (*S*)-CzACN b). The delayed spectra were measured at 77 K. The spectrum in blue is the delayed emission of binaphthol in solid state.

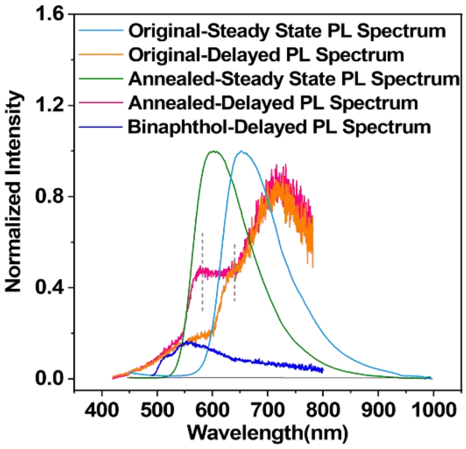

Fig. 33 Steady-state and delayed PL spectra for the pristine and annealed samples of (*R*,*S*)-CzACN. The delayed spectra were measured at 77 K. The spectrum in blue is the delayed emission of binaphthol in solid state.

The phosphorescence spectra for the pristine and annealed samples of the enantiomers and racemate were recorded, and the results were presented in Figs. S32 and S33. In each delayed emission spectrum, there is a broad band at around 555 nm, which can be assigned to the phosphorescence of binaphthalene.[2] It means that the delayed luminescence of the samples at cryogenic temperature was probably composed of phosphorescence from both the binaphthalene unit and the donor-acceptor fragment of the involved molecules. In fact, the donor-acceptor fragments of (*R*)-CzACN and (*S*)-CzACN emitted very weak phosphorescence even at 77 K. Therefore, the phosphorescence bands of the binaphthalene moieties became observable in the delayed spectra of the samples and partially overlapped with those of the donor-acceptor fragments. As a result, it is difficult to accurately determine the ΔE_{ST} values of (*R*)-CzACN and (*S*)-CzACN from experimental data. Herein, by using the onset of steady-state PL spectra and the first emission peak of phosphorescence from the donor-acceptor fragments, the ΔE_{ST} values for the pristine and annealed samples of the enantiomers were roughly estimated to be 0.17 eV and 0.11 eV, while those of the racemate were evaluated to be 0.16 eV and 0.18 eV, respectively. All of them are smaller than 0.20 eV, indicating that the samples have potential to produce TADF. However, the experimental result was that no delayed emission could be observed from the pristine cocrystals of racemate. Consequently, the unstable triplet excited states resulted from their lower energy levels and the relatively active molecular motions, which are supported by the reduce of $\Phi_{F,s}$ value, may be responsible for the loss of TADF property of the red cocrystal.

8 ^{1}H NMR Spectra, ^{13}C NMR Spectra, and Mass Spectra

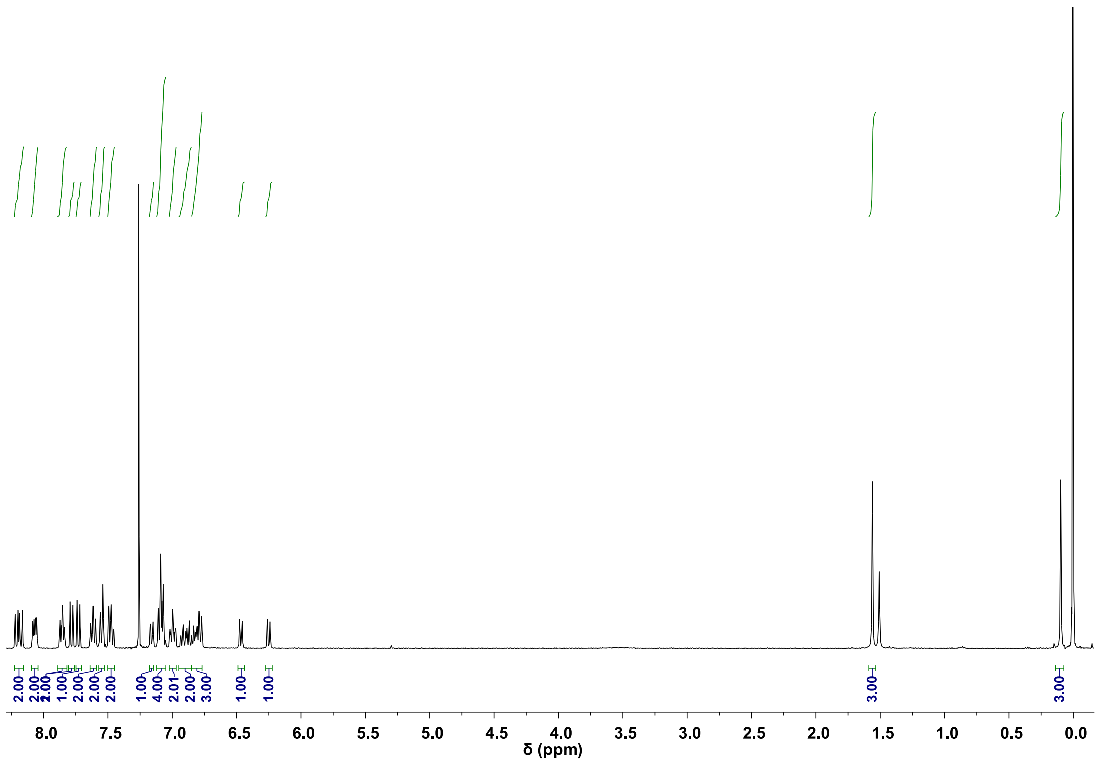

Fig. S34 ^{1}H NMR spectrum of (*R*)-CzACN in $CDCl_3$.

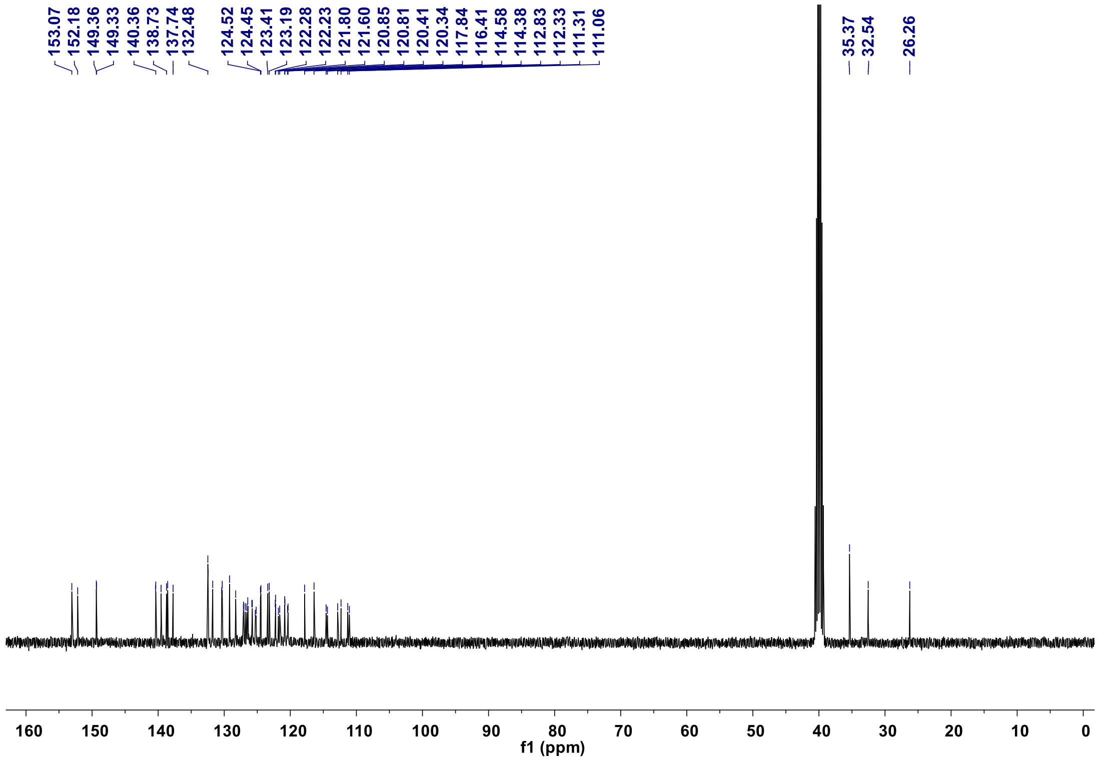

Fig. S35 ^{13}C NMR spectrum of (*R*)-CzACN in DMSO-d_6.

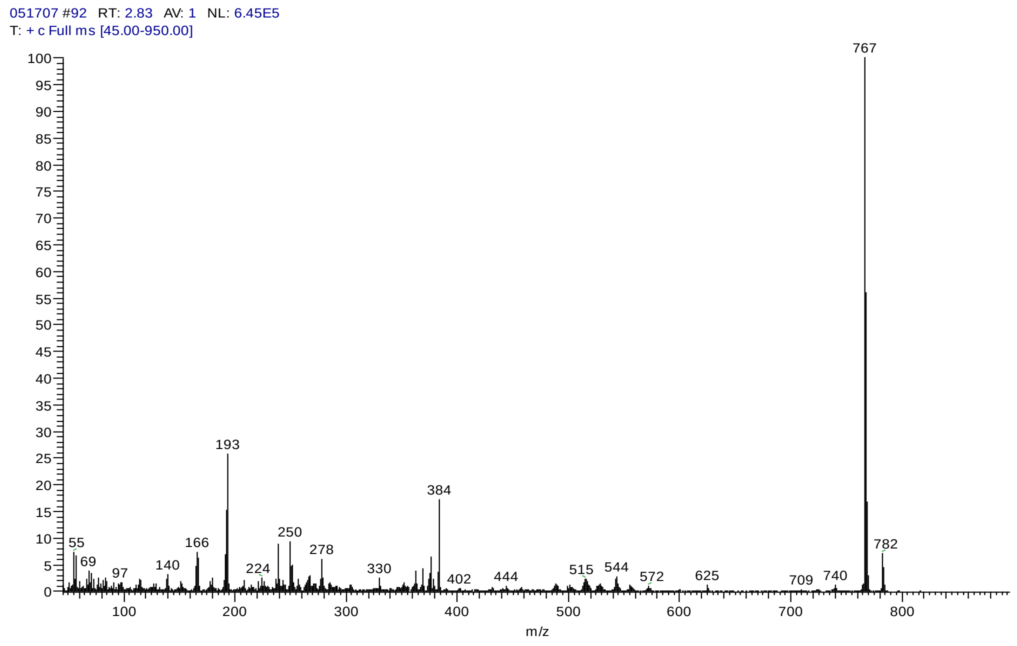

Fig. S36 EI-MS of (*R*)-CzACN.

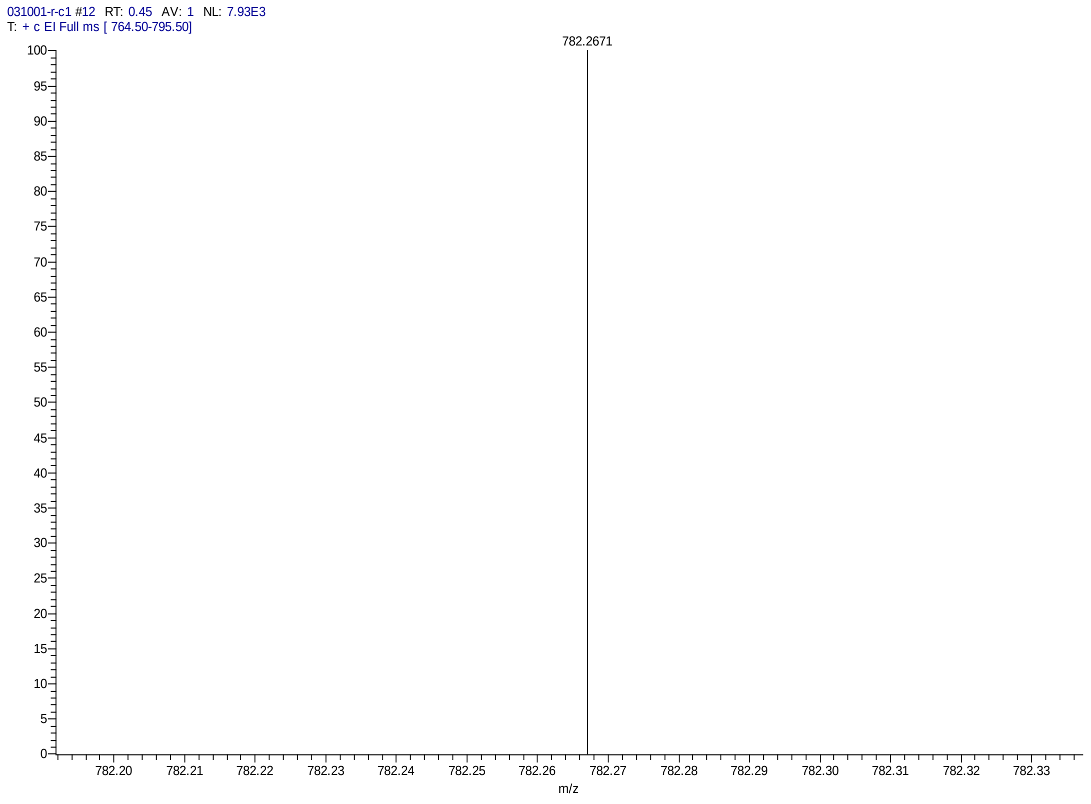

Fig. S37 HRMS of (*R*)-CzACN.

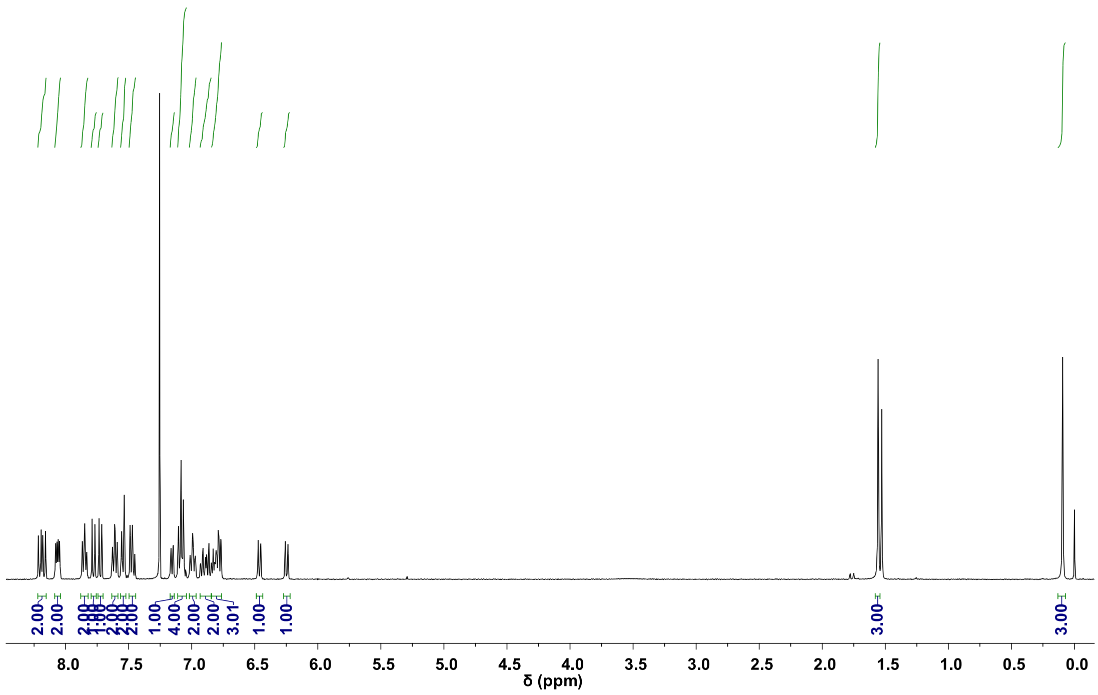

Fig. S38 ^{1}H NMR spectrum of (*S*)-CzACN in $CDCl_3$.

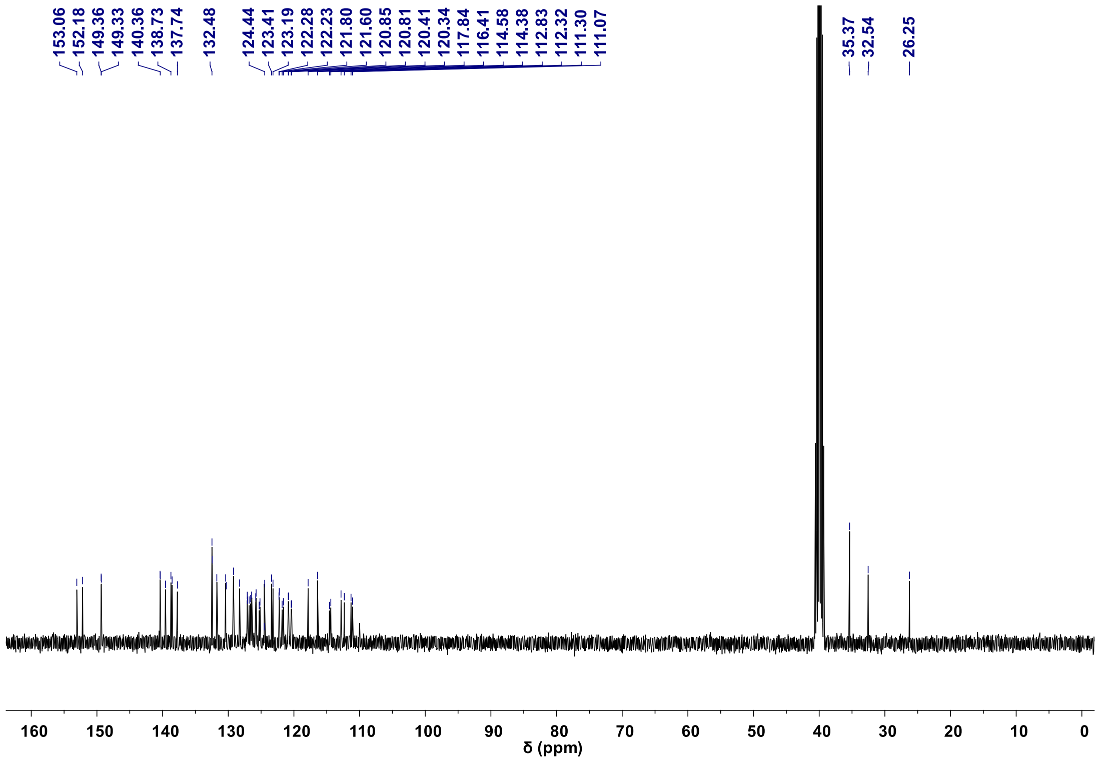

Fig. S39 ^{13}C NMR spectrum of (*S*)-CzACN in DMSO-d_6.

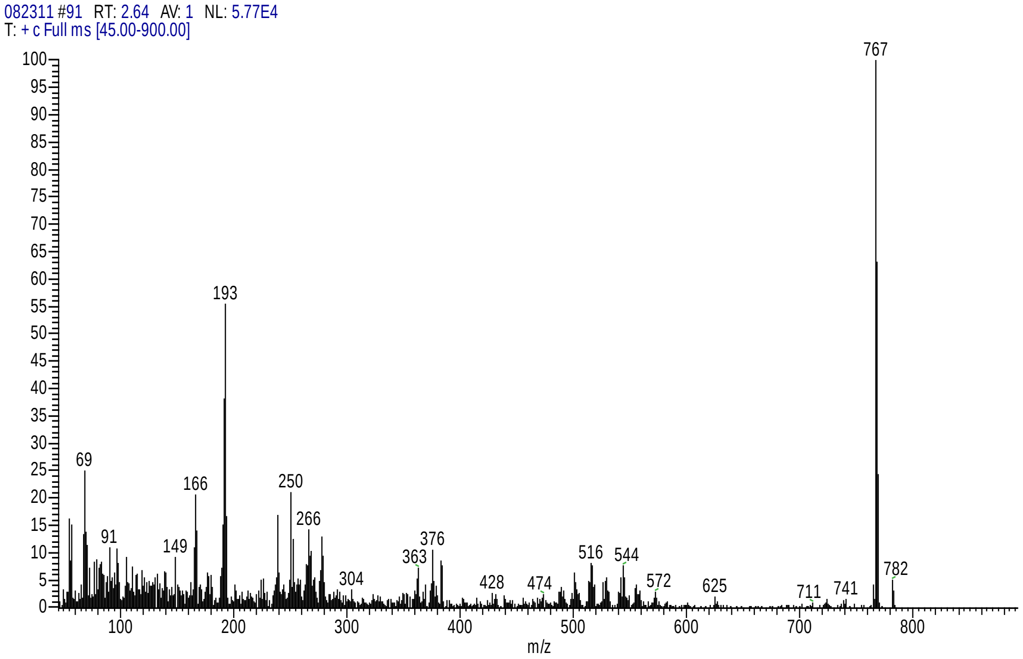

Fig. S40 EI-MS of (*S*)-CzACN.

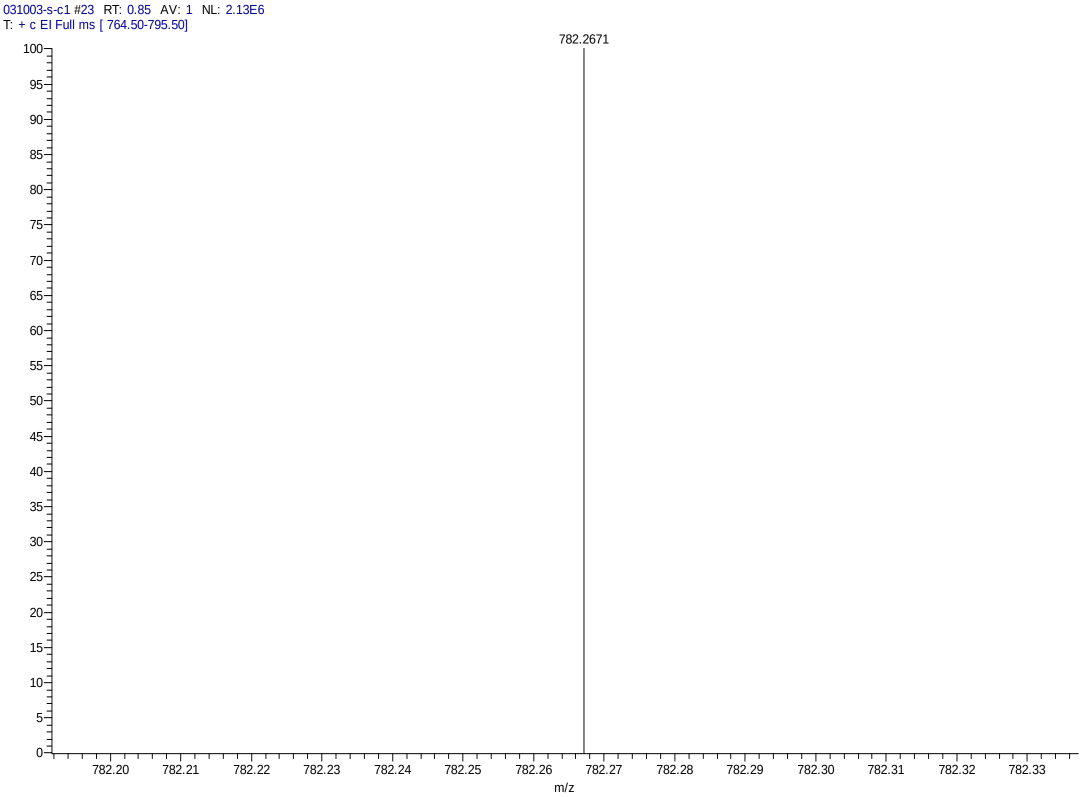

Fig. S41 HRMS of (*S*)-CzACN.

9 Data for the Single Crystal Structures

Table S1. Crystal data and structure refinement for the single crystals

Compound	(*S*)-CzACN-SC_{eo}	(*S*)-CzACN-SC_{er}	(*S*)-CzACN-SC_{rr}
formula	$C_{55}H_{34}N_4O_2$	$C_{55}H_{34}N_4O_2$	$C_{55}H_{34}N_4O_2$
fw	782.2682	782.2682	782.2682
crystal system,	monoclinic	orthorhombic	monoclinic
T / K	150(2)	293.3(4)	149(2)
space group	$P2_1$	$P2_12_12_1$	$P2_1/c$
a/Å	9.0078(8)	10.9114(3)	25.1331(13)
b /Å	35.009(3)	16.3200(5)	8.9596(5)
c/Å	14.5108(14)	26.1644(9)	19.2248(11)
α/ °	90	90	90
β/ °	90.134(6)	90	93.183(3)
γ/ °	90	90	90
V/Å^3, Z	4576.1(7), 4	4659.2(2), 4	4322.4(4), 4
F (000)	1968	1824	1800
crystal size / mm^3	0.19×0.02×0.01	0.90×0.04×0.02	0.20×0.08×0.05
reflections collected / unique (R_{int})	86355/19970 (R_{int} = 0.0930)	14493/9084 (R_{int} = 0.0504)	49979/9594 R_{int} = 0.0713)
obsd reflns [$I \geq 2\sigma(I)$]	11081	7002	6537
data/restraints/parameter	19970/81/1213	9084/0/608	9594/0/579
D_c/ Mg·m^{-3}	1.383	1.242	1.334
μ/ mm^{-1}	1.814	0.622	1.134
goodness-of-fit on F^2	1.081	1.055	1.051
R_1,[a] wR_2[b] [$I \geq 2\sigma(I)$]	0.1057, 0.2142	0.0624, 0.1539	0.1095, 0.2747
R_1, wR_2 (all data)	0.1783, 0.2484	0.0793, 0.1758	0.1460, 0.2956

[a] $R_1 = \Sigma||F_o| - |F_c||/\Sigma|F_o|$. [b] $wR_2 = [\Sigma[w(F_o^2 - F_c^2)^2]/\Sigma w(F_o^2)^2]^{1/2}$, where $w = 1/[^2(F_o)^2 + (aP)^2 + bP]$ and $P = (F_o^2 + 2F_c^2)/3$

10 References

[1] M. J. Frisch, G. W. Trucks, H. B. Schlegel, G. E. Scuseria, M. A. Robb, J. R. Cheeseman, G. Scalmani, V. Barone, G. A. Petersson, H. Nakatsuji, X. Li, M. Caricato, A. V. Marenich, J. Bloino, B. G. Janesko, R. Gomperts, B. Mennucci, H. P. Hratchian, J. V. Ortiz, A. F. Izmaylov, J. L. Sonnenberg, Williams, F. Ding, F. Lipparini, F. Egidi, J. Goings, B. Peng, A. Petrone, T. Henderson, D. Ranasinghe, V. G. Zakrzewski, J. Gao, N. Rega, G. Zheng, W. Liang, M. Hada, M. Ehara, K. Toyota, R. Fukuda, J. Hasegawa, M. Ishida, T. Nakajima, Y. Honda, O. Kitao, H.

Nakai, T. Vreven, K. Throssell, J. A. Montgomery Jr., J. E. Peralta, F. Ogliaro, M. J. Bearpark, J. J. Heyd, E. N. Brothers, K. N. Kudin, V. N. Staroverov, T. A. Keith, R. Kobayashi, J. Normand, K. Raghavachari, A. P. Rendell, J. C. Burant, S. S. Iyengar, J. Tomasi, M. Cossi, J. M. Millam, M. Klene, C. Adamo, R. Cammi, J. W. Ochterski, R. L. Martin, K. Morokuma, O. Farkas, J. B. Foresman, D. J. Fox, Wallingford, CT, 2016.

[2] L. Gu, W. Ye, X. Liang, A. Lv, H. Ma, M. Singh, W. Jia, Z. Shen, Y. Guo, Y. Gao, H. Chen, D. Wang, Y. Wu, J. Liu, H. Wang, Y. X. Zheng, Z. An, W. Huang and Y. Zhao, *J. Am. Chem. Soc.*, 2021, DOI: 10.1021/jacs.1c08118.
